# Activity and Thermal Stability of Cobalt(II)-Based Olefin Polymerization Catalysts Adorned with Sterically Hindered Dibenzocycloheptyl Groups

**DOI:** 10.3390/molecules24102007

**Published:** 2019-05-25

**Authors:** Muhammad Zada, Liwei Guo, Yanping Ma, Wenjuan Zhang, Zygmunt Flisak, Yang Sun, Wen-Hua Sun

**Affiliations:** 1 Key Laboratory of Engineering Plastics and Beijing National Laboratory for Molecular Science, Institute of Chemistry, Chinese Academy of Sciences, Beijing 100190, China; mzada17@iccas.ac.cn (M.Z.); mrliwei_guo@163.com (L.G.); myanping@iccas.ac.cn (Y.M.); sy0471103@iccas.ac.cn (Y.S.); 2 CAS Research/Education Center for Excellence in Molecular Sciences, University of Chinese Academy of Sciences, Beijing 100049, China; 3 Beijing Key Laboratory of Clothing Materials R&D and Assessment, Beijing Engineering Research Center of Textile Nanofiber, School of Materials Science and Engineering, Beijing institute of Fashion Technology, Beijing 100029, China; 4 Faculty of Chemistry, University of Opole, Oleska 48, 45-052 Opole, Poland

**Keywords:** Dibenzocycloheptyl group, 2,6-bis(imino)pyridylcobalt(II) chloride precatalysts, thermal stability, high molecular-weight saturated/unsaturated polyethylene

## Abstract

Five examples of unsymmetrical 2-(2,4-bis(dibenzocycloheptyl)-6-methylphenyl- imino)ethyl)-6-(1-(arylyimino)ethyl)pyridine derivatives (aryl **=** 2,6-Me_2_C_6_H_3_ in **L1**; 2,6-Et_2_C_6_H_3_ in **L2**; 2,6-i-Pr_2_C_6_H_3_ in **L3**; 2,4,6-Me_3_C_6_H_2_ in **L4** and 2,6-Et_2_-4-MeC_6_H_2_ in **L5**) were prepared and characterized. Treatment with CoCl_2_ offered the corresponding cobalt precatalysts **Co1**–**Co5**, which were characterized by FT-IR and NMR spectroscopy as well as elemental analysis. The molecular structures of **Co3** and **Co4** determined by single crystal X-ray diffraction revealed distorted square pyramidal geometries with τ_5_ values of 0.052–0.215. Activated with either MAO or MMAO, the precatalysts displayed high activities in ethylene polymerization, where **Co1** with the least bulky substituents exhibited a peak activity of 1.00 **×** 10^7^ g PE mol^−1^ (Co) h^−1^ at 60 °C. With MAO as a cocatalyst, the activity was reduced only by one order of magnitude at 90 °C, which implies thermally stable active sites. The polymerization product was highly linear polyethylene with vinyl end groups. **Co3** with the most sterically hindered active sites was capable of generating polyethylene of high molecular weight, reaching 6.46 **×** 10^5^ g mol^−1^. Furthermore, high melting point and unimodal molecular weight distribution were observed in the resulting polyethylene. It must be stressed that the thermal stability of the catalyst and the molecular weight of the obtained polyethylene attain the highest values reported for the unsymmetrical 2,6-bis(imino)pyridylcobalt (II) chloride precatalysts.

## 1. Introduction

**Scheme 1:**
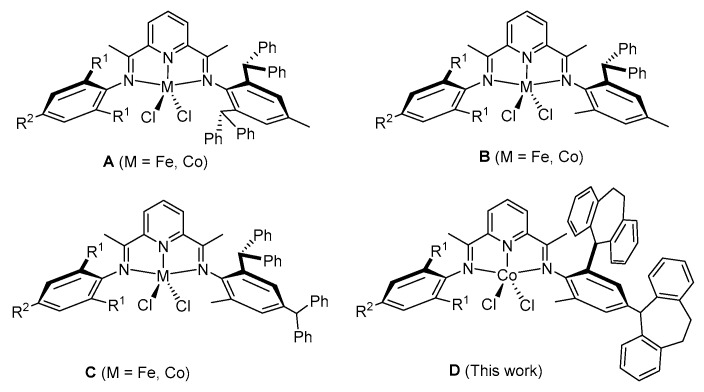
**A**–**D**: Structural variations in the bis(imino)pyridine-iron and cobalt precatalysts.

The effective design of late transition metal precatalysts for ethylene polymerization and oligomerization has attracted considerable attention over the last twenty years. The contributing factors are the ease of preparation and capability to generate diverse polymers, including highly linear and highly branched polyethylenes with varied contents of unsaturated groups, as well as waxes [1,2,3,4,5]. The initial reports on the bis(imino)pyridyliron(II) or cobalt(II) chloride precatalysts in ethylene polymerization [1,2] were fundamental to subsequent investigations focusing on the relationship between the structure, productivity and thermal stability of precatalysts. In these works, selection of the metal and variations in the ligand framework were carried out in the catalyst design phase and determined the physical properties and applications of the polymer [6,7]. For example, in contrast to branched polyethylenes generated by Ni(II) or Pd(II) systems [1,2,6,7], highly linear polyethylene is usually obtained by using Fe(II) or Co(II) catalysts. Successive modifications of the substituents attached to the existing bis(imino)pyridine framework aimed at improving the catalytic performance and thermal stability of the corresponding metal complexes [1,2,6,7,8] and usually modulated steric and electronic properties of the ligand [1,2,9,10,11,12,13,14,15,16,17,18]. As a result, novel iron and cobalt precatalysts bearing the bis(imino)pyridine framework were developed [8,9,10,11,12,13,14,15,16,17,18,19,20], in which the cycloalkyl rings were fused with the pyridine moiety [21,22,23,24,25,26,27,28,29,30,31,32,33,34,35]. In particular, the benzhydryl group which exerts significant steric hindrance, combined with the electron withdrawing or the electron donating substituents led to highly active and thermally stable precatalysts (Scheme 1, **A**) [36,37,38,39,40,41,42] that surpass the systems reported before [1,2]. The precatalysts bearing the ligands with the benzhydryl groups at the ortho-positions along with the electron withdrawing substituents at the para- position in one of the aryl rings attached to the imine nitrogen atom exhibited good thermal stability at the expense of slightly lower activity [43]. Within this series of precatalysts, fluorinated benzhydryl groups offered the highest thermal stability [44]. The incorporation of a single benzhydryl substituent at the *ortho-* position in one of the aryl rings attached to the imine nitrogen atom together with the electron donating methyl substituents at the remaining *ortho-* and *para-* positions in the 2,6-bis(imino)pyridine ligand improved the activity of the iron precatalyst with respect to the cobalt system (Scheme 1, **B**) [39]. Moreover, adding another benzhydryl substituent at the *para-*position within the same aryl group enhanced the catalytic performance and thermal stability of 2,6-bis(imino)pyridine-based iron and cobalt precatalysts, and influenced the molecular weight of polyethylene (Scheme 1, **C**) [38,45]. The corresponding precatalysts with the fluorinated benzhydryl substituents have also been investigated in ethylene polymerization, displaying pronounced activity and enhanced thermal stability [46]. In addition to that, cobalt bis(imino)pyridine precatalysts bearing the 2,4-dibenzhydrylnaphthyl moiety attached to the imino nitrogen atom generate highly linear polyethylene with the vinyl end groups [13,41]. In contrast to the unsymmetrically substituted systems listed above, the 2,6-bis(imino)pyridyliron or cobalt precatalysts bearing two benzhydryl substituents attached to each aryl rings offer enhanced thermal stability, but the activity in ethylene polymerization is somewhat compromised [47].

Recently, our research group has focused on the ligands, where single and double carbocyclic units of different size were fused with the pyridine ring, and synthesized the corresponding cobalt, iron and chromium complexes in order to investigate the effect of these modifications on the catalytic performance, thermal stability and the properties of the resultant polyethylene [8,48,49,50].

In order to further refine our understanding of the action of bulky substituents on the thermal stability of precatalysts, polymer productivity and its molecular weight, we turned our attention to dibenzocycloheptyl substituents attached to one of the aryl rings. In this contribution, we disclose the ligand design with 2,4-bis(dibenzocycloheptyl)-6-methylphenylamine, which serves as a starting point to prepare the unsymmetrical 2-(1-(2,4-bis(dibenzocycloheptyl)-6-methylphenylimino)ethyl) -6-(1-(arylimino)ethyl)pyridine and the corresponding cobalt(II) chloride precatalysts (Scheme 1, **D**). The complete synthetic procedure and characterization of ligands together with the corresponding cobalt precatalysts, as well as the catalytic performance in ethylene polymerization, thermal stability and the properties of the resultant polymer are reported.

## 2. Results

### 2.1. Synthesis and Characterization

**Scheme 2:**
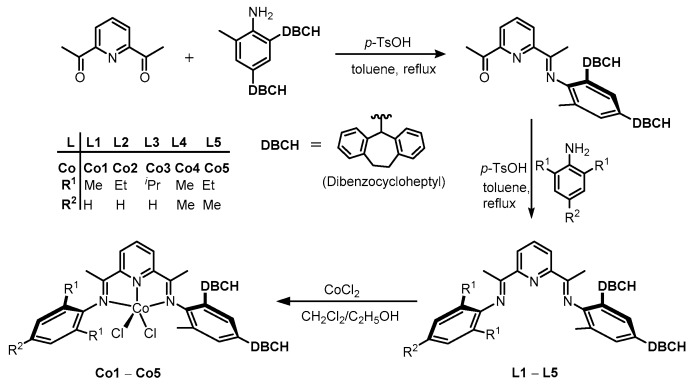
Synthesis of ligands **(L1**–**L5)** and the corresponding cobalt(II) chloride complexes **(Co1**–**Co5)**.

2,4-bis(Dibenzocycloheptyl)-6-methylaniline is prepared in a good yield according to the literature method [51,52,53,54,55]. The condensation reaction with one molar equivalent of 2,6-diacetylpyridine generates the monoketone derivative, 2-acetyl-6-(1-(2,4-bis(dibenzocyclo- heptyl)-6-methylphenylimino)ethyl)pyridine. Subsequent reactions with the corresponding anilines lead to a series of 2-(1-(2,4-bis(dibenzocycloheptyl)-6-methylphenylimino)ethyl)-6-(1-(arylimino)- ethyl)pyridine ligands (**L1**–**L5**); {aryl = 2,6-Me_2_C_6_H_3_ (**L1**), 2,6-Et_2_C_6_H_3_ (**L2**), 2,6-*^i^*Pr_2_C_6_H_3_ (**L3**), 2,4,6-Me_3_C_6_H_2_ (**L4**) and 2,6-Et_2_-4-MeC_6_H_2_ (**L5**)}. Upon treatment with CoCl_2_, the corresponding precatalysts **Co1**–**Co5** are obtained (Scheme 2). 

All the organic compounds and cobalt precatalysts mentioned above were characterized by FT-IR spectroscopy, NMR spectroscopy and elemental analysis. The representative complexes **Co3** and **Co4** were the subject of single-crystal X-ray diffraction analysis. In the FT-IR spectra of the complexes, the wavenumbers corresponding to the stretching vibrations of the C=N_imine_ bonds observed in the range of 1624–1625 cm^−1^ are lower, comparing with the respective ligands (1640–1649 cm^−1^). This finding reveals the effective coordination of the metal center and the ligand donor atoms, as reported earlier [36,46]. The ^1^H-NMR spectra of the cobalt precatalysts were recorded in deuterated dichloromethane CD_2_Cl_2_ at ambient temperature (see Supporting Information, Figures S1–S5). The characteristic peaks were assigned by comparison with the spectra of related bis(imino)pyridyl precatalysts [18,56,57]. Each spectrum reveals the unsymmetrical nature; for example, **Co1** shows two distinct signals for the m-pyridyl protons, which appear downfield with the chemical shifts of 113.26 and 110.26 ppm, respectively; each with the relative area of 1 and the *p*-pyridyl proton with the chemical shift of 37.57 ppm and the area of 1. Moreover, the peaks for the methyl groups attached to the aryl rings at the *ortho-*position were observed upfield with chemical shifts of −25.35 to −29.44 ppm and the acetyl protons downfield at 1.22 to 1.54 ppm, respectively; each with an area of 3. The remaining spectra are similar; for example, in **Co4** and **Co5**, distinct peaks are observed for the methyl protons attached to the aryl ring at the para-position with the chemical shifts of 17.56 and 18.63 ppm, respectively. The elemental analysis of all ligands and complexes was consistent with their formulae. In addition, the molecular structure of **Co3** and **Co4** were determined by single-crystal X-ray diffraction analysis.

### 2.2. X-Ray Crystallographic Studies

**Figure 1:**
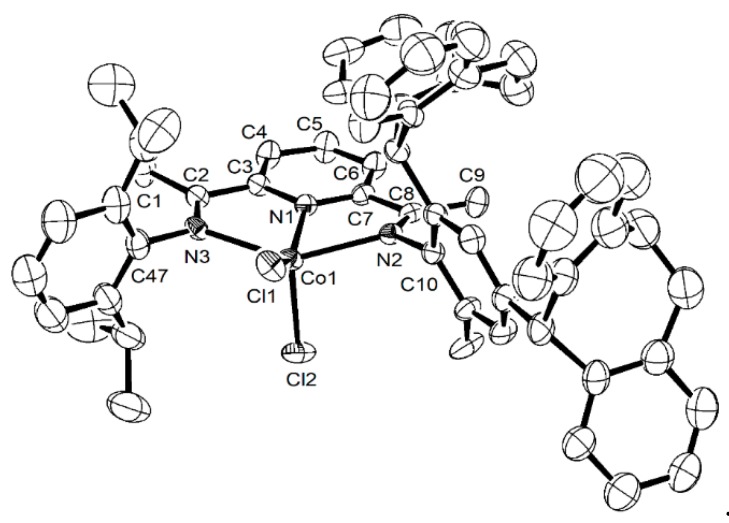
ORTEP diagram of **Co3** with thermal ellipsoids set at the probability level of 30%. All hydrogen atoms and another molecule of the complex have been omitted for clarity.

**Figure 2:**
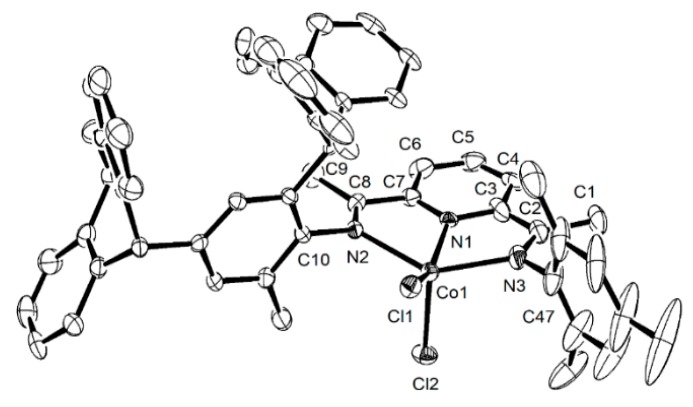
ORTEP diagram of **Co4** with thermal ellipsoids set at the probability level of 30%. All hydrogen atoms and another molecule of the complex have been omitted for clarity.

Single crystals of **Co3** and **Co4** suitable for the X-ray determinations were grown by the slow diffusion of diethyl ether into a solution of the corresponding complex in dichloromethane. During structural refinement two independent molecules (***A*** and ***B***) were obtained; only molecule ***A*** is displayed in Figure 1 and Figure 2. The selected bond lengths and angles are collected in Table 1 (for molecule ***A***) and in Table S1 (for molecule ***B***). The complexes comprise a single cobalt center bound to the 2,6-(bisarylimino)pyridine chelating ligand and two halide ligands, resulting in a distorted square pyramidal geometry. This geometry is further rationalized by the tau value (*τ*_5_) which is defined by the following equation:

*τ*_5_ = (*β* – *α*)/60, (1)

where *β* is the largest angle and *α* is the second largest angle in the coordination sphere (Table 2); a perfect square pyramid and a perfect trigonal bipyramid are indicated by the tau values of zero and one, respectively [58,59,60]. The nitrogen atoms (N1, N2 and N3) and one chlorine atom Cl(1) form the basal plane and the second chlorine atom Cl(2) occupies the apical position. The cobalt atoms lie out of the N1, N2, N3 plane in each complex with the dihedral angles of 89.14–88.53° (***A***), and 89.56–88.98° (***B***). Similar observations have been reported for other bis(imino)pyridine complexes [36,37,38,39,40,41,42,43,44,45,46]. Different substitution patterns of both aryl rings attached to the imine nitrogen atoms and varied steric hindrance around this atom are reflected by the modest imbalance in the corresponding Co–N_imine_ bond lengths, e.g., 2.214(5) Å vs. 2.228(5) Å in **Co3** (form A). In both **Co3** and **Co4**, the Co–N_pyridine_ bond is always stronger than any of the Co–N_imine_ bonds [36,37,38,39,40,41,42,46]. The N(2)–C(8) bond lengths in both complexes reveal the typical features of a C=N bond.

### 2.3. Evaluation of Cocatalyst

Previous studies on iron and cobalt bis(iminopyridine) catalysts indicated that methyl- aluminoxane (MAO) and modified methylaluminoxane (MMAO) are more effective cocatalysts compared with alkylaluminum reagents in ethylene polymerization [11,12,36,37,38,39,40,41,42]. In order to investigate the catalytic performance of the cobalt precatalysts toward ethylene polymerization, **Co1** was selected as the precatalyst for initial screening and the reaction conditions such as temperature, Al/Co ratio and time were systematically varied under ethylene pressure of 10 atm using methylaluminoxane (MAO) or modified methylaluminoxane (MMAO) as cocatalysts. Then the catalytic performance of the remaining cobalt precatalysts **Co2**–**Co5** was investigated at the conditions established before. Additionally, the catalytic activity of precatalyst **Co1** at ethylene pressure of 5 and 1 atm was also determined. The molecular weight (*M*_w_) and molecular weight distribution (*M*_w_/*M*_n_) of the resultant polyethylene were ascertained by gel permeation chromatography and the melting temperature was measured by differential scanning calorimetry (DSC). The microstructure of typical polyethylene samples was analyzed by the high-temperature NMR (^1^H/^13^C) spectroscopy. 

#### 2.3.1. Ethylene Polymerization with (**Co1**–**Co5**)/MAO

**Figure 3:**
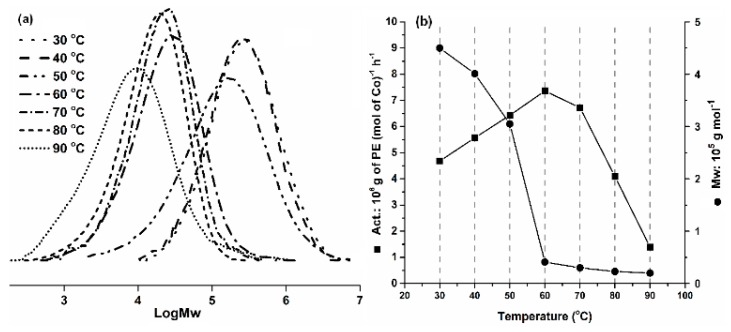
GPC curves of the obtained polyethylene (**a**); activity and *M*_w_ as a function of reaction temperature (**b**) for the **Co1**/MAO system (Table 3, entries 1–7).

**Figure 4:**
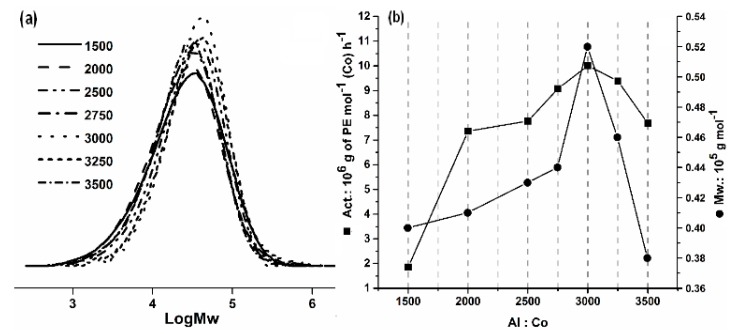
GPC curves of the obtained polyethylene (**a**); activity and *M*_w_ as a function of the Al/Co ratio (**b**) for the **Co1**/MAO system (Table 3, entries 4 and 8–13).

The results of polymerization tests carried out for **Co1** in toluene under ethylene pressure of 10 atm are summarized in Table 3. The polymerization runs were conducted at the temperature varied between 30 and 90 °C with the Al/Co ratio fixed at 2000 over the reaction time of 30 min (Table 3, entries 1–7); the dependence of the catalytic activity and the polyethylene molecular weight on the reaction temperature is plotted in Figure 3, which shows that the activity gradually increases with the temperature and achieves the highest value of 7.36 × 10^6^ g of PE (mol of Co)^−1^ h^−1^ at 60 °C (Table 3, entry 4). The catalytic activity is still high at 70 °C, reaching 6.72 × 10^6^ g of PE (mol of Co)^−1^ h^−1^ and amounting to 4.10 × 10^6^ g of PE (mol of Co)^−1^ h^−1^ at 80 °C (Table 3, entries 5, 6). Beyond that point, the activity sharply decreases to 1.40 × 10^6^ g of PE (mol of Co) ^−1^ h^−1^ at 90 °C (Table 3, entry 7). These observations indicate thermally stable active sites and the decrease in catalytic activity can be ascribed to the lower solubility of ethylene or partial deactivation of active species at high temperature [9,10,11,12,36,37,38,39,40,41,42,43,44,45,46,57,61]. High melting point of the resultant polyethylene (*T*_m_ = 130.7–135.6 °C) indicates its linear structure; this fact is further confirmed by the high-temperature NMR (^1^H/^13^C) spectroscopy. Moreover, polyethylene molecular weight decreases from 4.50 × 10^5^ g mol^–1^ to 0.20 × 10^5^ g mol^–1^ when the polymerization temperature is raised from 30 °C to 90 °C; this could be attributed either to the low solubility of ethylene monomer in toluene or the increased rates of chain transfer reactions at elevated temperature, or both (Figure 3) [36,37,38,39,40,41,42,43,44,45,46,57,61]. The effect of the Al/Co ratio ranging from 1500 to 3500 on the catalytic activity was studied at the optimized reaction temperature of 60 °C. The activity increases with the growing Al/Co ratio, reaching 10.01 × 10^6^ g of PE (mol of Co)^−1^ h^−1^ at Al/Co = 3000 (Table 3, entry 11). Beyond that point the activity drops down to 7.68 × 10^6^ g of PE (mol of Co)^−1^ h^−1^ at Al/Co = 3500 (Table 3, entry 13). The molecular weight of polyethylene gradually increases from 0.40 × 10^5^ g mol^–1^ to 0.52 × 10^5^ g mol^–1^ when the Al/Co ratio is raised from 1500 to 3000 (Table 3, entries 4, 8–11) and then sharply decreases to 0.38 × 10^5^ g mol^–1^ (Table 3, entry 13)–see also Figure 4. This decrease could be attributed to the chain migration and termination reactions occurring at higher concentration of the cocatalyst [33,62]. The polyethylene *T*_m_ values range from 132.1 °C to 132.9 °C (Table 3, entries 4, 8–13). With the optimum values of the reaction temperature (60 °C) and the Al/Co ratio (3000), the effect of reaction time ranging from 5 to 60 min on the polymerization process was explored (Table 3, entries 11, 14–17).

**Figure 5:**
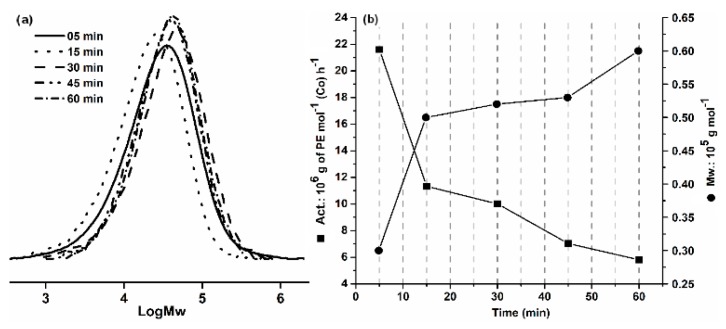
GPC curves of the obtained polyethylene (**a**); activity and *M*_w_ as a function of reaction time (**b**) for the **Co1**/MAO system (Table 3, entries 11 and 14–17).

The catalytic activity is inversely related to the polymerization time, and the highest value of 21.60 × 10^6^ g of PE (mol of Co) ^−1^ h^−1^ was found at 5 min run time, indicating a short induction time required to generate the active sites (Table 3, entry 14). With the prolonged reaction time the activity gradually decreases (Table 3, entries 4 and 14–17) and the moderate value of 5.81 × 10^6^ g of PE (mol of Co) ^−1^ h^−1^ was found even for 60 min (Table 3, entry 17), suggesting rather long lifetime of the active sites. The molecular weight of the obtained polyethylene increases constantly with reaction time. The product exhibits unimodal molecular weight distribution and the melting point in the range of 132.1–132.8 °C. The plot of activity and the molecular weight of polyethylene as a function of the reaction time is given in Figure 5. Similar dependencies have been observed for the related catalytic systems [36,37,38,39,40,41,42,43,44,45,46].

**Figure 6:**
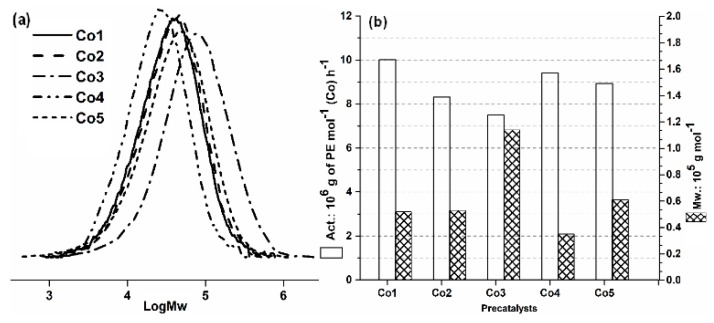
GPC curves of the obtained polyethylene (**a**); activity and *M*_w_ for different precatalysts at the optimized reaction conditions (**b**)—see Table 4, entries 1–5.

**Figure 7:**
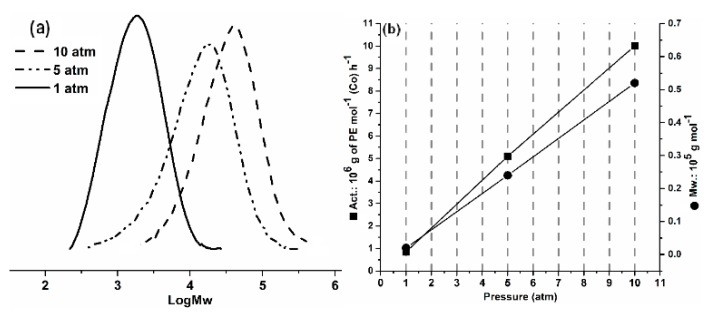
GPC curves of the obtained polyethylene (**a**); activity and *M*_w_ as a function of ethylene pressure (**b**) for the **Co1**/MAO system at the optimized reaction conditions (Table 3, entries 11, 18 and 19).

The performance of the remaining cobalt precatalysts (**Co2**–**Co5**) in ethylene polymerization was investigated at the optimum reaction conditions established for the **Co1**/MAO system, i.e., the reaction temperature of 60 °C, the Al/Co ratio of 3000, ethylene pressure of 10 atm and the run time of 30 min–see Table 4, entries 1 – 5. Steric effects have impact on the overall catalytic activity, which decreases in the following order: **Co1** > **Co4** > **Co5** > **Co2** > **Co3**; thus the highest activity of 10.01 × 10^6^ g of PE (mol of Co)^−1^ h^−1^ is attained by the **Co1** precatalyst with the least bulky substituent R^1^; likewise **Co3** bearing relatively bulky R^1^ displays the lowest activity of 7.49 × 10^6^ g of PE (mol of Co)^−1^ h^−1^. The values of activity and the polyethylene molecular weight for **Co1**–**Co5** are given in Figure 6. The advantageous effect of bulky R^1^ substituent on the polyethylene molecular weight is clearly seen for **Co3** (Table 4, entry 3), where the polymer of relatively high molecular weight is generated (Table 4, entry 3). This indicates that bulky substituents protect the active sites and suppress the chain transfer. Similar observations were reported in the literature [33,36,38,39,42,45]. The polyethylene obtained using the **Co1**–**Co5** precatalysts displays high melting point (*T_m_*) of 132.3–135.7 °C (Table 4, entries 1–5); this finding is also in good agreement with the previous reports on bis(imino)pyridine catalysts [36,37,38,39,40,41,42,43,44,45,46]. In order to examine the effect of ethylene pressure on the catalytic activity and the molecular weight of the product, further polymerization tests were run at the optimized reaction conditions (T = 60 °C, Al/Co = 3000 and t = 30 min) at 5 and 1 atm (Table 3, entries 18 and 19). The results indicating strong influence of pressure on both quantities are shown in Figure 7. In the case of polyethylene, unimodal molecular weight distribution and the melting points (*T_m_*) of 131.4 and 123.1 °C were attained for p = 5 and p = 1 atm, respectively.

#### 2.3.2. Ethylene Polymerization with (**Co1**–**Co5**)/MMAO

The data for ethylene polymerization catalyzed with the **Co1**/MMAO system are shown in Table 5 in a similar manner as in the case of the **Co1**/MAO system. Typical ethylene polymerization runs were performed in toluene at the Al/Co ratio fixed at 2000, under 10 atm ethylene pressure and over reaction time of 30 min. Initially, the reaction temperature was varied from 30 to 90 °C (Table 5, entries 1–7 and Figure S6), and the gradual increase in the catalytic activity reaching the maximum of 6.28 × 10^6^ g of PE (mol of Co)^−1^ h^−1^ at 50 °C was observed (Table 5, entry 3). Beyond that point the activity drops, which can be attributed to the partial deactivation of active species or lower solubility of ethylene at elevated temperature [36,37,38,39,40,41,42,43,44,45,46,57,61]; but the relatively high value of 2.99 × 10^6^ g of PE (mol of Co)^−1^ h^−1^ is maintained at 80 °C and 1.89 × 10^6^ g of PE (mol of Co)^−1^ h^−1^ is still attainable even at 90 °C (Table 5, entry 7). Again, unprecedented thermal stability was recorded for the **Co1**/MMAO system, which–however – does not surpass the **Co1**/MAO catalyst described in the previous section. As a result of increased polymerization temperature, the molecular weight of polyethylene decreases from 3.80 to 0.25 × 10^5^ g mol^–1^ (Table 5, entries 1–7). The impact of temperature on the activity and molecular weight shown in Figure S6 indicates unimodal molecular weight distributions. In the next step, the Al/Co molar ratio was varied from 1000 to 3000 and the polymerization was carried out at the optimized temperature of 50 °C (Table 5, entries 3 and 8–13). The catalytic activity first increases, reaching the maximum value of 7.89 × 10^6^ g of PE (mol of Co)^−1^ h^−^^1^ at the Al/Co ratio of 1500 (Table 5, entry 10), and then decreases (Table 5, entries 11–13; see also Figure S7).

The deactivation observed at higher Al/Co ratios may be ascribed to more frequent events of chain transfer from cobalt to aluminium [33,62,63]. Then, the stability of active sites was studied under optimized reaction temperature of 50 °C and the Al/Co molar ratio of 1500 for the reaction time from 5 to 60 min (Table 5, entries 10, 14–17). The catalytic activity is again inversely related to the reaction time–the highest value of 28.80 × 10^6^ g of PE (mol of Co) ^−1^ h^−1^ was found for the 5 min run time, which indicates a short induction time necessary to generate the active sites (Table 5, entry 14). After that, the catalytic activity steadily decreases on prolonging the polymerization time (Table 5, entries 15–17) and the lowest activity of 5.76 × 10^6^ g of PE (mol of Co) ^−1^ h^−1^ is found for the polymerization time of 60 min (Table 5, entry 17). These observations are similar to those made for the **Co1**/MAO system and agree with the previous reports on bis(imino)pyridine catalysts [36,37,38,39,40,41,42,43,44,45,46]. The catalyst still retains remarkable activity after 60 min, which suggests relatively long lifetime of the active sites. Moreover, the molecular weight of the obtained polyethylene steadily increases with the reaction time. The high melting point (*T_m_*) of 135.3–135.6 °C and unimodal molecular weight distributions are the typical features of the obtained polyethylene (Figure S8).

The catalytic performance of the remaining precatalysts **Co2**–**Co5** was studied in the optimized polymerization conditions determined for the **Co1**/MMAO system (Al/Co = 1500, T = 50 °C, p = 10 atm and t = 30 min); see Table 6, entries 1–5. The **Co1** precatalyst with the lowest steric hindrance around the active site exhibits the highest activity of 7.89 × 10^6^ g of PE (mol of Co)^−1^ h^−1^ (Table 6, entry 1) and the overall catalytic activity decreases in the following order: **Co1 > Co4 > Co2 > Co5 > Co3,** which–by analogy to other cobalt precatalysts [36,38,39,42,45]–indicates the pronounced effect of R^1^ on the activity (see Figure S9). It must be mentioned that the precatalysts activated with MMAO generate polyethylene of much higher molecular weight (Table 6, entry 3), compared with the systems containing MAO (Table 4, entry 3) [33,36]. Ethylene pressure has also marked effect on the catalytic activity and the polyethylene molecular weight (Figure S10); the trends are similar to those observed for the **Co1**/MAO system.

### 2.4. Polyethylene Microstructural Properties

Relatively high value of melting point (132.3–135.7 °C) for the majority of polyethylene samples obtained at different reaction conditions indicates highly linear macromolecules. More detailed investigation of the product microstructure was carried out by means of high-temperature ^1^H- and ^13^C-NMR spectroscopy for the representative sample obtained with **Co1**/MAO at 60 °C (Table 3, entry 11). The spectra were recorded in 1,1,2,2-teterachloroethane-*d* at 100 °C and interpreted according to literature method [38,39,45,64,65,66]. 

**Figure 8:**
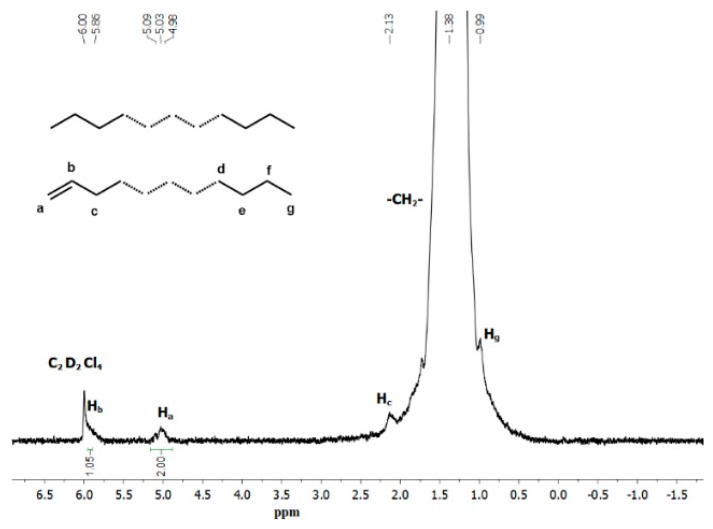
^1^H-NMR spectrum of the polyethylene obtained with **Co1**/MAO (Table 3, entry 11).

**Figure 9:**
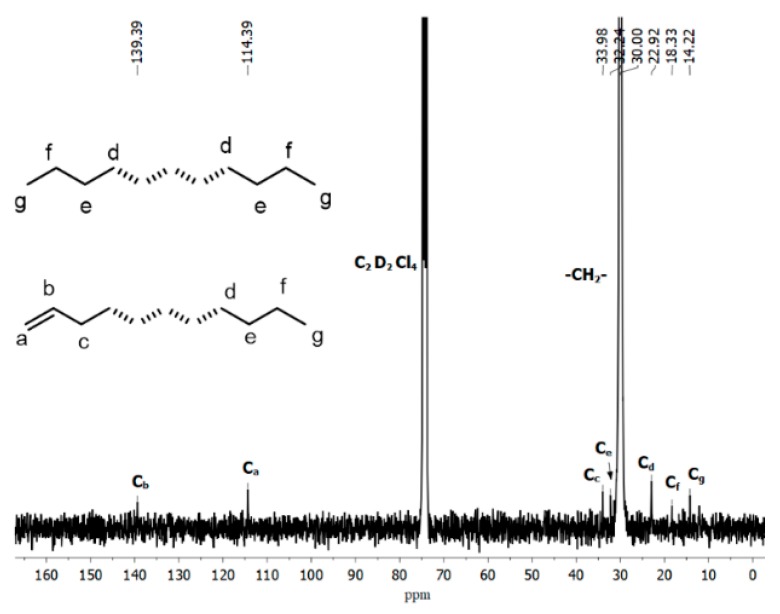
^13^C-NMR spectrum of the polyethylene obtained with **Co1**/MAO (Table 3, entry 11)

The ^1^H-NMR spectrum (Figure 8) reveals the coexistence of polyethylene bearing both saturated and unsaturated end groups. However, the exact ratio of both polymer types is unclear due to overlapping peaks. Vinyl end groups (–CH=CH_2_) were identified as two multiplets around 5.86 and 5.03 ppm with the relative peak area of 1 and 2. High intensity singlet at around 1.38 ppm corresponding to the protons of the –(CH_2_)_n_– mers and another signal at 2.13 ppm due to the protons adjacent to the vinyl group (H_c_) were observed. The signal of the methyl group (H_g_) at 0.99 ppm overlaps with the –(CH_2_)_n_– band; therefore the exact ratio of unsaturated to saturated end groups cannot be determined. The NMR spectra suggest a highly linear structure of both saturated and unsaturated polyethylene. The presence of the vinyl end group was further confirmed by the ^13^C-NMR spectrum (Figure 9), in which two distinct peaks belonging to the vinyl carbon atoms located at the end of the polymer chain (C_a_ and C_b_) were detected at 114.39 and 139.39 ppm, together with the peak corresponding to the adjacent carbon atom (C_c_) at 33.93 ppm. The signal of the –(CH_2_)_n_– mers was recorded at 30.00 ppm. The carbon atoms of the methyl group at the saturated end of the macromolecule were observed at 14.22 ppm (C_g_), along with the carbon atoms located at the close vicinity (C_d_, C_e_, and C_f_) at 32.24, 22.92 and 18.33 ppm, respectively [38,39,45,64,65,66].

The high melting point of another set of samples corresponding to the polyethylene obtained with the **Co1**/MMAO system (131.3–136.2 °C) suggests highly linear saturated polymer (Table 5, entry 1–7). This was further confirmed by the high-temperature ^1^H-/^13^C-NMR spectra of the selected representative sample obtained at the conditions defined in Table 5, entry 10. The signals corresponding to the –(CH_2_)_n_– mers [38,39,45,64,65,66] observed in both spectra (Figs. S11 and S12) reveal highly linear and saturated polyethylene.

### 2.5. Comparison of the Current Precatalyst with Systems Reported Before

**Figure 10:**
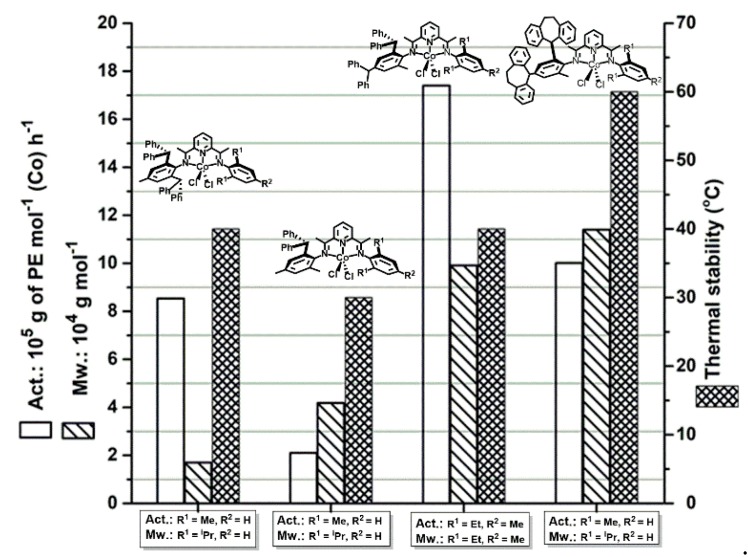
Comparison of the catalytic activity, polyethylene molecular weight and thermal stability of the selected precatalysts bearing benzhydryl groups with the current system at the optimized conditions with MAO or MMAO as cocatalysts and 10 atm of C_2_H_4_.

The comparison of the precatalyst investigated in this work with the previously reported cobalt systems [39,42,45] shown in Figure 10 reveals several interesting trends. The unprecedented thermal stability observed for the current system highlights the potential of the dibenzocycloheptyl substituent introduced in this work. Comparing with the catalysts reported before, polyethylene of the highest molecular weight was obtained due to the bulkiness that prevents chain transfer by protecting the active sites; this finding is consistent with the literature [33,36]. Interestingly, the catalytic activity is not impaired significantly and the current system is able to outperform several precatalysts with the benzhydryl substituents attached to the aryl rings [39,42].

## 3. Materials and Methods 

### 3.1. General Information

All manipulations involving air or moisture sensitive compounds were carried out using standard Schlenk techniques under inert nitrogen atmosphere. The solvents were distilled under a nitrogen atmosphere prior to use. Methylaluminoxane (MAO, 1.46 M in toluene) and modified methylaluminoxane (MMAO, 1.93 M in *n*-heptane) were purchased from Albemarle Corp. (Nanjing, China). High-purity ethylene was purchased from Beijing Yanshan Petrochemical Co. (Beijing, China) and used as received. All other reagents were purchased from Aldrich (Beijing, China), Acros (Beijing, China) or Beijing Chemicals (Beijing, China). 2,4-bis(Dibenzocycloheptyl)-6-methylaniline was prepared using the procedure reported in the literature [51,52,53,54,55]. The ^1^H- and ^13^C-NMR spectra of the free ligands and complexes were recorded on a DMX 400 MHz instrument (Bruker, Karlsruhe, Germany) at ambient temperature with TMS as an internal standard. The FT-IR spectra were recorded on a System 2000 FT-IR spectrometer (Perkin Elmer, Shanghai, China) and elemental analyses were determined using a Flash EA 1112 microanalyzer (Thermo Electron SPA, Beijing, China). The molecular weight (*M*_w_) and molecular weight distribution (*M*_w_/*M*_n_) of the polyethylene were determined using a PL-GPC220 instrument (Beijing, China) at 150 °C using 1,2,4-trichlorobenzene as a solvent. The polyethylene melting point was measured with differential scanning calorimetry (DSC, Q2000, TA Instruments, Beijing, China) under nitrogen atmosphere. A typical polyethylene sample of approximately 5.0 mg was heated up to 160 °C at a heating rate of 20 °C per minute and kept for 3 min at this temperature to remove its thermal history; then it was cooled to *−*20 °C at the rate of 20 °C per minute. For the ^13^C-NMR spectra of polyethylene, a weighed sample (90–100 mg) was mixed with 1,1,2,2-tetrachloroethane-*d*_2_ (2 mL) in a 5 mm standard glass tube; TMS was applied as an internal standard. Inverse-gated ^13^C spectra were recorded at 100 °C on a Bruker DMX 300 spectrometer at 75.47 MHz with the number of scans from 3934 to 3966. Operating conditions used: spectral width 17,985.6 kHz; acquisition time 1.8 s; relaxation delay 2.0 s and pulse width 15.5 µs.

### 3.2. Synthesis of 2-acetyl-6-{1-(2,4-bis(dibenzocycloheptyl)-6-methylphenylimino)ethyl}pyridine

A mixture of 2,6-diacetylpyridine (2.13 g, 13.10 mmol) and 2,4-bis(dibenzocycloheptyl)-6- methylaniline (6.42 g, 13.10 mmol) was added into toluene (100 mL) along with a catalytic amount of *p-*toluenesulfonic acid (15%). The mixture was refluxed for 10 h and upon completion of reaction (checked with TLC) the volatiles were evaporated at reduced pressure. The residue was purified by column chromatography (basic alumina); elution with petroleum ether/ethyl acetate (25:2) afforded a yellow powder (3.1 g, 37%). Mp: 201–203 ^o^C. FT-IR (KBr cm^−1^): 3050 (w), 3061 (w), 3013 (w), 2925 (w), 2831 (w), 2835 (w), 1703 (υ(C=O), s), 1630 (υ(C=N), m), 1609 (w), 1555 (w), 1519 (w), 1489 (m), 1454 (w), 1435 (w), 1354 (s), 1308 (m), 1238 (m), 1160 (w), 1124 (w), 1098 (w), 1071 (w), 1047 (w), 1019 (w), 995 (w), 947 (w), 920 (w), 882 (w), 842 (w), 812 (m), 792 (w), 758 (s), 738 (m), 705 (m), 677 (w). ^1^H- NMR (CDCl_3_, TMS): δ 8.60 (d, *J* = 8.04 Hz, 1H, Py–H), 8.13 (d, *J* = 7.60 Hz, 1H, Py–H), 7.97 (t, *J* = 7.80 Hz, 1H, Py–H), 7.20–6.77 (m, 15H, Ar–*H*), 6.62 (s, 1H, Ar–Hm), 6.50 (t, *J* = 7.20 Hz, 1H, Ar–H), 6.43 (s, 1H, Ar–Hm), 5.10 (s, 1H, –CH–), 4.94 (s, 1H, –CH–), 3.00–2.75 (m, 4H, –CH_2_–), 2.72 (s, 3H, –CH_3_), 2.65–2.60 (m, 4H, –CH_2_–), 1.80 (s, 3H, –CH_3_), 1.47 (s, 3H, –CH_3_). ^13^C-NMR (CDCl_3_, TMS): δ 200.2, 168.6, 155.3, 152.3, 145.6, 141.3, 141.2, 140.2, 140.0, 139.7, 139.2, 138.8, 138.4, 137.0, 131.4, 131.4, 131.2, 131.1, 131.0, 130.9, 130.4, 130.2, 129.4, 128.7, 127.2, 127.0, 126.9, 126.5, 126.4, 126.0, 125.9, 125.6, 125.4, 124.6, 124.6, 122.4, 57.8, 56.4, 32.6, 32.4, 31.9, 30.4, 25.6, 17.9, 15.9.

### 3.3. Synthesis of ligands **L1–L5**; 2-{2,4-(C_15_H_13_)_2_-6-MeC_6_H_2_N}-6-(ArN) C_9_H_9_N

#### 3.3.1. Ar = 2,6-Me_2_C_6_H_3_ (**L1**). 

2,6-Dimethylaniline (0.21 g, 1.70 mmol) was slowly added into the mixture of 2-acetyl-6- [1-(2,4-bis(dibenzocycloheptyl)-6-methylphenylimino)ethyl]pyridine (1.10 g, 1.70 mmol) and catalytic amount of *p*-toluenesulfonic acid (15%) in toluene (100 mL). The mixture was refluxed for 8 h using a Dean-Stark trap. Upon completion of the reaction (checked with TLC), the mixture was cooled to room temperature and then the volatiles were evaporated at reduced pressure. The residue was purified by (basic) alumina column chromatography; elution with petroleum ether/ethyl acetate (50:2) afforded **L1** as a yellow powder (0.45 g, 35%). Mp: 256–258 ^o^C. FT-IR (KBr cm^−1^): 3062 (w), 3017 (w), 2933 (w), 2883 (w), 2831 (w), 1643 (υ(C=N), s), 1572 (υ(C=N), w), 1491 (w), 1449 (s), 1363 (s), 1300 (w), 1249 (w), 1206 (m), 1164 (w), 1120 (m), 1097 (w), 1043 (w), 1026 (w), 991 (w), 967 (w), 942 (w), 882 (w), 811 (m), 773 (s), 747 (s), 710 (m). ^1^H-NMR (CDCl_3_, TMS): *δ* 8.47 (d, *J* = 7.60 Hz, 2H, Py–H), 7.94 (t*, J* = 7.80 Hz, 1H, Py–H), 7.20–6.82 (m, 18H, Ar–H), 6.62 (s, 1H, Aryl–H*_m_*), 6.55 (t, *J* = 7.02 Hz, 1H, Ar–H), 6.43 (s, 1H, Aryl–H*_m_*), 5.12 (s, 1H, –CH–), 4.97 (s, 1H, –CH–), 3.13–2.90 (m, 3H, –CH_2_–), 2.75–2.56 (m, 4H, –CH_2_–), 2.36–2.30 (m, 1H, –CH_2_–), 2.18 (s, 3H, –CH_3_), 2.11 (s, 3H, –CH_3_), 2.05 (s, 3H, –CH_3_), 1.83 (s, 3H, –CH_3_), 1.52 (s, 3H, –CH_3_). ^13^C-NMR (CDCl_3_, TMS): *δ* 169.1, 167.3, 155.0, 154.9, 148.8, 145.8, 141.3, 141.1, 140.2, 140.0, 139.8, 139.7, 139.3, 138.6, 138.5, 136.6, 131.5, 131.4, 131.2, 131.1, 130.9, 130.5, 130.3, 129.4, 128.7, 127.9, 127.1, 127.0, 126.9, 126.5, 126.3, 126.0, 125.9, 125.6, 125.5, 125.5, 124.7, 123.0, 122.3, 122.0, 57.8, 56.4, 32.5, 32.3, 31.9, 30.6, 17.9, 16.4, 16.0. Anal. calcd for C_54_H_49_N_3_ (739.99): C, 87.65; H, 6.67; N, 5.68. Found: C, 87.48; H, 6.67; N, 5.69.

#### 3.3.2. Ar = 2,6-Et_2_C_6_H_3_ (**L2**). 

Using a similar procedure as described for the synthesis of **L1**, **L2** was prepared as a yellow powder (0.34 g, 26%). Mp: 215–217 ^o^C. FT-IR (KBr cm^−1^): 3059 (w), 3015 (w), 2935 (w), 2876 (w), 2824 (w), 1643 (υ(C=N), s), 1569 (υ(C=N), w), 1489 (w), 1448 (m), 1363 (m), 1302 (w), 1242 (w), 1219 (w), 1197 (w), 1119 (m), 1100 (w), 1076 (w), 1050 (w), 1014 (w), 963 (w), 910 (w), 871 (w), 849 (w), 802 (w), 787 (w), 759 (s), 704 (w). ^1^H-NMR (CDCl_3_, TMS): *δ* 8.45 (d, *J* = 8.02 Hz, 2H, Py–H), 7.94 (t*, J* = 7.80 Hz, 1H, Py–H), 7.20–6.81 (m, 18H, Ar–H), 6.62 (s, 1H, Aryl–H*_m_*), 6.54 (t, *J* = 7.20 Hz, 1H, Ar–H), 6.43 (s, 1H, Aryl–H*_m_*), 5.11 (s, 1H, –CH–), 4.97 (s, 1H, –CH–), 3.01–2.89 (m, 3H, –CH_2_–), 2.74–2.57 (m, 5H, –CH_2_–), 2.50–2.30 (m, 4H, –CH_2_–), 2.19 (s, 3H, –CH_3_), 1.83 (s, 3H, –CH_3_), 1.51 (s, 3H, –CH_3_), 1.20 (t, *J* = 7.40 Hz, 3H, –CH_2_CH_3_), 1.14 (t, *J* = 7.40 Hz, 3H, –CH_2_CH_3_). ^13^C-NMR (CDCl_3_, TMS): *δ* 169.1, 167.0, 155.0, 154.9, 147.8, 145.9, 141.3, 141.2, 140.3, 140.0, 139.8, 139.7, 139.4, 138.6, 138.5, 136.6, 131.5, 131.4, 131.23, 131.12, 130.99, 130.52, 130.29, 129.44, 128.71, 127.14, 127.02, 126.87, 126.50, 126.30, 126.0, 125.9, 125.6, 124.7, 123.3, 122.3, 122.0, 57.8, 56.4, 32.5, 32.4, 31.9, 30.6, 24.7, 24.6, 17.9, 16.8, 16.1, 13.8, 13.7. Anal. calcd for C_56_H_53_N_3_ (768.04): C, 87.57; H, 6.96; N, 5.47. Found: C, 87.34; H, 7.04; N, 5.60.

#### 3.3.3. Ar = 2,6-*^i^*^-^Pr_2_C_6_H_3_ (**L3**). 

Using a similar procedure as described for the synthesis of **L1**, **L3** was prepared as a yellow powder (0.38 g, 28%). Mp: 245–247 ^o^C. FT-IR (KBr cm^−1^): 3062 (w), 3012 (w), 2960 (m), 2923 (w), 2866 (w), 1649 (υ(C=N), s), 1593 (υ(C=N), w), 1560 (w), 1514 (w), 1487 (w), 1458 (m), 1435 (w), 1385 (w), 1364 (m), 1326 (w), 1302 (w), 1242 (w), 1217 (w), 1188 (w), 1165 (w), 1117 (m), 1076 (w), 1049 (w), 1016 (w), 960 (w), 934 (w), 905 (w), 879 (w), 841 (w), 815 (w), 789 (w), 769 (m), 760 (m), 752 (s), 747 (s), 721 (w), 704 (w), 679 (w). ^1^H-NMR (CDCl_3_, TMS): *δ* 8.44 (d, *J* = 7.60 Hz, 2H, Py–H), 7.94 (t*, J* = 7.80 Hz, 1H, Py–H), 7.20–6.80 (m, 18H, Ar–H), 6.61 (s, 1H, Aryl–H*_m_*), 6.53 (t, *J* = 7.20 Hz, 1H, Ar–H), 6.43 (s, 1H, Aryl–H*_m_*), 5.10 (s, 1H, –CH–), 4.96 (s, 1H, –CH–), 3.09–2.56 (m, 9H, –CH_2_/–CH–), 2.35–2.31 (m, 1H, –CH_2_–), 2.20 (s, 3H, –CH_3_), 1.83 (s, 3H, –CH_3_), 1.51 (s, 3H, –CH_3_), 1.21 (d, *J* = 6.80 Hz, 6H, –CH(CH_3_)_2_), 1.15 (d, *J* = 6.80 Hz, 6H, –CH(CH_3_)_2_). ^13^C-NMR (CDCl_3_, TMS): *δ* 169.3, 167.3, 155.2, 155.0, 146.7, 146.0, 141.4, 141.3, 140.4, 140.2, 140.0, 139.9, 139.5, 138.8, 138.7, 136.7, 136.0, 135.9, 131.7, 131.5, 131.4, 131.2, 131.1, 131.1, 130.6, 130.4, 129.6, 128.8, 127.3, 127.1, 127.0, 126.6, 126.4, 126.1, 126.0, 125.8, 125.7, 124.8, 123.7, 123.1, 122.4, 122.2, 57.9, 56.6, 32.7, 32.5, 32.1, 30.7, 28.6, 28.4, 23.4, 23.3, 23.1, 18.1, 17.3, 16.2. Anal. calcd for C_58_H_57_N_3_ (796.09): C, 87.50; H, 7.22; N, 5.28. Found: C, 87.37; H, 7.16; N, 5.36.

#### 3.3.4. Ar = 2,4,6-Me_3_C_6_H_2_ (**L4**). 

Using a similar procedure as described for the synthesis of **L1**, **L4** was prepared as a yellow powder (0.42 g, 32%). Mp: 219–221 ^o^C. FT-IR (KBr cm^−1^): 3055 (w), 3012 (w), 2961 (w), 2891 (w), 2857 (w), 1640 (υ(C=N), s), 1566 (υ(C=N), w), 1515 (w), 1482 (s), 1450 (w), 1433 (w), 1382 (w), 1359 (m), 1307 (w), 1283 (w), 1253 (w), 1217 (s), 1182 (w), 1121 (m), 1075 (w), 1043 (w), 1012 (w), 972 (w), 942 (w), 910 (w), 886 (w), 851 (s), 813 (w), 787 (w), 749 (s), 709 (w), 678 (w). ^1^H-NMR (CDCl_3_, TMS): *δ* 8.48 (d, *J* = 7.60 Hz, 2H, Py–H), 7.94 (t*, J* = 7.80 Hz, 1H, Py–H), 7.21–6.83 (m, 17H, Ar–H), 6.63 (s, 1H, Aryl–H*_m_*), 6.60 (t, *J* = 7.20 Hz, 1H, Ar–H), 6.44 (s, 1H, Aryl–H*_m_*), 5.11 (s, 1H, –CH–), 4.98 (s, 1H, –CH–), 3.14–2.91 (m, 3H, –CH_2_–), 2.74–2.58 (m, 5H, –CH_2_–), 2.32 (s, 3H, –CH_3_), 2.18 (s, 3 H, –CH_3_), 2.10 (s, 3H, –CH_3_), 2.02 (s, 3H, –CH_3_), 1.83 (s, 3H, –CH_3_), 1.52 (s, 3H, –CH_3_). ^13^C-NMR (CDCl_3_, TMS): *δ* 169.2, 167.7, 155.3, 155.0, 146.4, 146.0, 141.4, 141.2, 140.4, 140.1, 140.0, 139.8, 139.5, 138.7, 138.7, 136.7, 132.3, 131.7, 131.5, 131.4, 131.2, 131.1, 130.6, 130.4, 129.6, 128.8, 128.7, 127.3, 127.1, 127.0, 126.6, 126.4, 126.1, 126.0, 125.8, 125.7, 125.4, 125.4, 124.9, 122.0, 122.1, 58.6, 57.9, 56.7, 32.7, 32.5, 32.0, 30.7, 20.9, 18.6, 18.1, 18.0, 17.9, 16.5, 16.2. Anal. calcd for C_55_H_51_N_3_ (754.01): C, 87.61; H, 6.82; N, 5.57. Found: C, 87.28; H, 6.84; N, 5.37.

#### 3.3.5. Ar = 2,6-Et_2_-4-MeC_6_H_2_ (**L5**). 

Using a similar procedure as described for the synthesis of **L1**, **L5** was prepared as a yellow powder (0.75 g, 54%). Mp: 236–238 ^o^C. FT-IR (KBr cm^−1^): 3024 (w), 2968 (w), 2939 (w), 2878 (w), 2826 (w) 1647 (υ(C=N), s), 1567 (υ(C=N), w), 1522 (w), 1488 (w), 1457 (s), 1420 (w), 1409 (w), 1366 (m), 1333 (w), 1307 (w), 1277 (w), 1244 (w), 1214 (s), 1177 (w), 1141 (w), 1117 (m), 1076 (w), 1046 (w), 995 (w), 972 (w), 936 (w), 910 (w), 886 (w), 859 (s), 829 (m), 792 (m), 759 (s), 737 (w), 703 (w), 665 (w). ^1^H-NMR (CDCl_3_, TMS): *δ* 8.45 (d, *J* = 8.04 Hz, 2H, Py–H), 7.94 (t*, J* = 7.80 Hz, 1H, Py–H), 7.21–6.82 (m, 17H, Ar–H), 6.63 (s, 1H, Aryl–H*_m_*), 6.55 (t, *J* = 7.20 Hz, 1H, Ar–H), 6.44 (s, 1H, Aryl–H*_m_*), 5.11 (s, 1H, –CH–), 4.98 (s, 1H, –CH–), 3.13–2.90 (m, 3H, –CH_2_–), 2.75–2.56 (m, 4H, –CH_2_–), 2.47–2.40 (m, 4H, –CH_2_–), 2.38 (s, 3H, –CH_3_), 2.36–2.26 (m, 1H, –CH_2_–), 2.19 (s, 3 H, –CH_3_), 1.84 (s, 3H, –CH_3_), 1.52 (s, 3H, –CH_3_), 1.20 (t, *J* = 7.60 Hz, 3H, –CH_2_CH_3_), 1.14 (t, *J* = 7.40 Hz, 3H, –CH_2_CH_3_). ^13^C-NMR (CDCl_3_, TMS): *δ* 169.3, 167.4, 155.3, 155., 146.0, 145.4, 141.4, 141.3, 140.4, 140.1, 140.0, 139.8, 139.5, 138.7, 138.7, 136.7, 132.5, 131.7, 131.5, 131.4, 131.2, 131.1, 130.6, 130.4, 129.5, 128.8, 127.2, 127.1, 127.0, 126.9, 126.8, 126.6, 126.4, 126.1, 126.0, 125.8, 125.7, 124.9, 122.3, 122.1, 57.9, 56.6, 32.7, 32.5, 32.0, 30.7, 24.8, 24.7, 21.1, 18.6, 18.1, 16.9, 16.2, 14.0, 13.9. Anal. calcd for C_57_H_55_N_3_ (782.02): C, 87.54; H, 7.09; N, 5.37. Found: C, 87.06; H, 7.30; N, 5.34.

### 3.4. Synthesis of complexes **Co1–Co5**; [2-{2,4-(C_15_H_13_)_2_-6-MeC_6_H_2_N}-6-(ArN) C_9_H_9_N]CoCl_2_

#### 3.4.1. **Co1** (Ar = 2,6-Me_2_C_6_H_3_). 

**L1** (0.20 g, 0.27 mmol) and CoCl_2_ (0.035 g, 0.27 mmol) were loaded into a Schlenk tube, followed by the addition of dichloromethane (5 mL) and ethanol (5 mL). The mixture was stirred at room temperature for 12 h and then the volatiles were evaporated at reduced pressure to give concentrated solution. The complex was precipitated using diethyl ether (20 mL), filtered, washed with diethyl ether (3 × 15 mL) and dried under reduced pressure to afford **Co1** as a green powder 0.17 g (71%). FT-IR (KBr cm^−1^): 3056 (w), 3013 (w), 2927 (w), 2902 (w), 2871 (w), 1624 (υ(C=N), w), 1588 (υ(C=N), m), 1490 (m), 1466 (m), 1447 (m), 1430 (m), 1368 (m), 1311 (w), 1260 (m), 1212 (m), 1162 (w), 1128 (w), 1101 (w), 1028 (w), 978 (w), 944 (w), 917 (w), 883 (w), 840 (w), 814 (m), 790 (w), 760 (s), 737 (m), 704 (m). ^1^H-NMR (CD_2_Cl_2_, TMS): *δ* 113.26 (1H, Py-H*_m_*), 110.26 (1H, Py-H*_m_*), 37.57 (1H, Py-H*_p_*), 33.45 (1H, –CH–), 27.93 (1H, Ar-H), 16.33 (1H, –CH–), 9.42 (1H, Ar-H), 8.81 (1H, Ar-H), 8.53 (1H, Ar-H), 8.12 (1H, Ar-H), 7.38 (1H, Ar-H), 7.14–7.03 (1H, Ar-H), 6.76 (1H, Ar-H), 6.12 (1H, Ar-H), 5.77 (1H, Ar-H), 5.41 (1H, Ar-H), 4.32–4.11 (4H, –CH_2_–), 2.17–1.97 (1H, Ar-H), 1.17–1.15 (4H, –CH_2_–), 0.31 (1H, Ar-H), 0.17 (1H, Ar-H), *−*1.22 (3H, –CH_3_), −1.54 (3H, –CH_3_), −1.80 (1H, Ar-H*_m_*), −3.73 (1H, Ar-H*_m_*), −4.54 (1H, Ar-H), −5.19 (1H, Ar-H), −8.45 (1H, Ar-H*_m_*), −13.43 (1H, Ar-H*_m_*), −14.12 (1H, Ar-H*_p_*), −25.35 (3H, –CH_3_), −26.42 (3H, –CH_3_), −29.44 (3H, –CH_3_). Anal. calcd for C_54_H_49_Cl_2_CoN_3_ (868.26): C, 74.56; H, 5.68; N, 4.83. Found: C, 75.23; H, 5.73; N, 4.86.

#### 3.4.2. **Co2** (Ar = 2,6-Et_2_C_6_H_3_). 

Using the same procedure as described for the synthesis of **Co1**, **Co2** was obtained as a green powder (0.15 g, 63%). FT-IR (KBr cm^−1^): 3060 (w), 3015 (w), 2962 (w), 2929 (w), 2874 (w), 2835 (w), 1625 (υ(C=N), w), 1587 (υ(C=N), m), 1491 (m), 1446 (m), 1437 (m), 1368 (m), 1315 (w), 1258 (m), 1211 (m), 1200 (w), 1130 (w), 1102 (w), 1027 (w), 976 (w), 944 (w), 918 (w), 878 (w), 811 (m), 791 (w), 763 (s), 739 (m), 705 (m). ^1^H-NMR (CD_2_Cl_2_, TMS): *δ* 113.42 (1H, Py-H*_m_*), 111.18 (1H, Py-H*_m_*), 41.27 (1H, Py-H*_p_*), 35.41 (1H, –CH–), 28.45 (1H, Ar-H), 16.21 (1H, –CH–), 10.20 (1H, Ar-H), 9.75 (1H, Ar-H), 9.62 (1H, Ar-H), 8.87 (1H, Ar-H), 8.25 (1H, Ar-H), 7.59 (1H, Ar-H), 6.88 (1H, Ar-H), 6.35 (2H, Ar-H), 5.93–5.78 (4H, –CH_2_–), 4.29 (1H, Ar-H), 2.86 (1H, Ar-H), 1.87 (1H, Ar-H), 1.63 (1H, Ar-H), 1.34–0.72 (4H, –CH_2_–), 0.42 (3H, –CH_3_), 0.06 (3H, –CH_3_), −1.86 (1H, Ar-H*_m_*), −3.42 (1H, Ar-H*_m_*), −5.23 (1H, Ar-H), −6.57 (1H, Ar-H), −6.41 (1H, Ar-H*_m_*), −11.62 (1H, Ar-H*_m_*), −12.37 (1H, Ar-H*_p_*), −17.02 (3H, –CH_3_), −20.34 (3H, –CH_3_), −26..52 (3H, –CH_3_), −36.20–−41.91 (4H, –CH_2_–). Anal. calcd for C_56_H_53_Cl_2_CoN_3_ (897.88): C, 74.91; H, 5.95; N, 4.68. Found: C, 74.86; H, 5.86; N, 4.67.

#### 3.4.3. **Co3** (Ar = 2,6-^i-^Pr_2_C_6_H_3_). 

Using the same procedure as described for the synthesis of **Co1**, **Co3** was obtained as a green powder (0.16 g, 64%). FT-IR (KBr cm^−1^): 3063 (w), 3015 (w), 2963 (w), 2929 (w), 2872 (w), 2834 (w), 1624 (υ(C=N), w), 1587 (υ(C=N), m), 1491 (m), 1463 (m), 1440 (m), 1368 (m), 1315 (w), 1261 (m), 1217 (w), 1161 (w), 1134 (w), 1102 (w), 1048 (w), 1027 (w), 977 (w), 942 (w), 918 (w), 881 (w), 840 (w), 812 (w), 792 (w), 763 (s), 749 (s), 709 (m), 652 (m). ^1^H-NMR (CD_2_Cl_2_, TMS): *δ* 115.71 (1H, Py-H*_m_*), 113.06 (1H, Py-H*_m_*), 48.23 (1H, Py-H*_p_*), 38.18 (1H, –CH–), 36.40 (1H, Ar-H), 18.39 (1H, –CH–), 13.36 (1H, Ar-H), 11.45 (1H, Ar-H), 10.55 (1H, Ar-H), 9.67 (1H, Ar-H), 8.74 (1H, Ar-H), 7.55 (2H, Ar-H), 7.15 (1H, Ar-H), 6.76 (1H, Ar-H), 6.18–5.91 (4H, –CH_2_–), 4.69 (1H, Ar-H), 3.60 (1H, Ar-H), 3.19 (3H, Ar-H), 2.57 (1H, –CH–), 1.90 (1H, –CH–), 1.33–0.83 (4H, –CH_2_–), −1.23 (1H, Ar-H*_m_*), −2.37 (3H, –CH_3_), −2.86 (3H, –CH_3_), −3.27 (3H, –CH_3_), −5.23 (1H, Ar-H*_m_*), −7.03 (1H, Ar-H*_m_*), −9.10 (1H, Ar-H*_m_*), −11.56 (1H, Ar-H), −13.45 (3H, –CH_3_), −14.56 (1H, Ar-H*_p_*), −24.03 (3H, –CH_3_), −26.77 (3H, –CH_3_), −31.49 (3H, –CH_3_). Anal. calcd for C_58_H_57_Cl_2_CoN_3_ (925.93): C, 75.23; H, 6.26; N, 4.54. Found: C, 74.96; H, 6.18; N, 4.49.

#### 3.4.4. **Co4** (Ar = 2,4,6-Me_3_C_6_H_2_).

Using the same procedure as described for the synthesis of **Co1**, **Co4** was obtained as a green powder (0.16 g, 67%). FT-IR (KBr cm^−1^): 3055 (w), 3014 (w), 2960 (w), 2923 (w), 2875 (w), 2834 (w), 1624 (υ(C=N), w), 1587 (υ(C=N), m), 1489 (m), 1437 (m), 1368 (m), 1313 (w), 1260 (m), 1217 (m), 1158 (w), 1130 (w), 1101 (w), 1026 (w), 978 (w), 943 (w), 917 (w), 880 (w), 847 (w), 808 (w), 762 (s), 749 (s), 705 (m). ^1^H-NMR (CD_2_Cl_2_, TMS): *δ* 112.64 (1H, Py-H*_m_*), 110.65 (1H, Py-H*_m_*), 38.06 (1H, Py-H*_p_*), 33.50 (1H, –CH–), 27.51 (1H, Ar-H), 17.56 (3H*_p_*, –CH_3_), 15.94 (1H, –CH–), 9.41 (1H, Ar-H), 8.85–8.76 (4H, –CH_2_–), 8.07 (1H, Ar-H), 7.40 (1H, Ar-H), 6.75 (1H, Ar-H), 6.16 (1H, Ar-H), 5.79 (1H, Ar-H), 5.46 (1H, Ar-H), 4.58–4.44 (2H, Ar-H), 4.18 (1H, Ar-H), 2.23 (1H, Ar-H), 1.32–1.15 (4H, –CH_2_–), 0.42 (1H, Ar-H), 0.04 (1H, Ar-H), *−*0.89 (3H, –CH_3_), *−*1.09 (3H, –CH_3_), *−*1.72 (1H, Ar-H*_m_*), *−*3.70 (1H, Ar-H*_m_*), *−*4.93 (1H, Ar-H), *−*5.20 (1H, Ar-H), *−*7.45 (1H, Ar-H*_m_*), *−*12.95 (1H, Ar-H*_m_*), *−*24.36 (3H, –CH_3_), *−*25.57 (3H, –CH_3_), *−*28.79 (3H, –CH_3_). Anal. calcd for C_55_H_51_Cl_2_CoN_3_ (883.85): C, 74.74; H, 5.82; N, 4.75. Found: C, 74.73; H, 5.84; N, 4.58.

#### 3.4.5. **Co5** (Ar = 2,6-Et_2_-4-MeC_6_H_2_). 

Using the same procedure as described for the synthesis of Co1, Co5 was obtained as a green powder (0.17 g, 68%). FT-IR (KBr cm^−1^): 3053 (w), 3014 (w), 2963 (w), 2927 (w), 2874 (w), 2834 (w), 1624 (υ(C=N), w), 1587 (υ(C=N), m), 1491 (m), 1452 (m), 1368 (m), 1315 (w), 1260 (m), 1215 (m), 1156 (w), 1127 (w), 1100 (w), 1027 (w), 978 (w), 943 (w), 916 (w), 880 (w), 857 (w), 811 (m), 763 (s), 750 (s), 704 (m). ^1^H-NMR (CD_2_Cl_2_, TMS): *δ* 112.95 (1H, Py-H*_m_*), 111.60 (1H, Py-H*_m_*), 41.33 (1H, Py-H*_p_*), 35.50 (1H, –CH–), 27.84 (1H, Ar-H), 18.63 (3H*_p_*, –CH_3_), 16.03 (1H, –CH–), 10.08 (1H, Ar-H), 9.75 (2H, Ar-H), 8.72 (1H, Ar-H), 8.17 (1H, Ar-H), 7.65 (1H, Ar-H), 6.82 (2H, Ar-H), 6.40–6.23 (4H, –CH_2_–), 5.90–5.58 (4H, –CH_2_–), 4.21 (1H, Ar-H), 2.82 (1H, Ar-H), 1.75–1.04 (3H, Ar-H), 0.70 (3H, –CH_3_), 0.49 (3H, –CH_3_), *−*0.16 (1H, Ar-H*_m_*), *−*3.45 (1H, Ar-H*_m_*), *−*5.63 (1H, Ar-H), *−*6.87 (1H, Ar-H), *−*7.51 (1H, Ar-H*_m_*), *−*12.62 (1H, Ar-H*_m_*), *−*17.06 (3H, –CH_3_), *−*20.66 (3H, –CH_3_), *−*24.84 (3H, –CH_3_), *−*32.25–*−*44.93 (4H, –CH_2_–). Anal. calcd for C_57_H_55_Cl_2_CoN_3_ (911.91): C, 75.07; H, 6.08; N, 4.61. Found: C, 75.01; H, 6.16; N, 4.57.

### 3.5. X-Ray Crystallographic Studies

Single crystal X-ray diffraction analysis of **Co3** and **Co4** was carried out on a Saturn 724+ CCD diffractometer (Rigaku, Tokyo, Japan) with the graphite-monochromated Mo-Kα radiation (λ = 0.71073 Å) at 173(2) K and cell parameters were obtained by global refinement of the positions of all collected reflections. Intensities were corrected for Lorentz and polarization effects, and an empirical absorption. The structures were solved by direct methods and refined by full-matrix least-squares on *F^2^*. All hydrogen atoms were placed in calculated positions. Structural solution of each complex and refinement were performed by SHELXT [67,68]. In the structural solution of each crystal, two identical structures were found. The solvent molecules which have no effect on the geometry of the main compound were also processed by using SHELXT [67,68]. All hydrogen atoms and one identical molecule of the complex have been omitted in the ORTEP diagrams for clarity. Crystal data and processing parameters for **Co3** and **Co4** are summarized in Table 7.

### 3.6. General Procedure for Ethylene Polymerization under 5/10 atm Pressure

Ethylene polymerization is conducted in a stainless-steel autoclave (250 mL) equipped with the temperature and pressure control system and a mechanical stirrer. The autoclave is initially evacuated and then filled with nitrogen gas. This process is repeated three times and after the final evacuation ethylene is introduced. A solution of the corresponding complex (1.5 μmol) in freshly distilled toluene (25 mL) is injected into the autoclave. Another batch of freshly distilled toluene (25 mL) is added and then the required amount of a cocatalyst (MAO, or MMAO) is injected. After adding another batch of toluene (50 mL), the autoclave is pressurized immediately with ethylene (10 atm) and the contents is stirred at a rate of 400 rpm. Upon completion, the stirring is stopped and the pressure is slowly released. The reaction is quenched with 10% hydrochloric acid in ethanol, and the polymer is washed with ethanol, filtered and dried under reduced pressure at 40 °C. Finally, the product is weighed. Schlenk tube is used instead of autoclave for ethylene polymerization at 1 atm, following similar procedure.

## 4. Conclusions

The successful incorporation of dibenzocycloheptyl groups at the 2- and 4-positions of one of the aryl rings attached to the imine nitrogen atom in the generic bis(imino)pyridine yielded an unsymmetrical species, which was further modified through variations in the second aryl group, leading to the **L1**–**L5** ligands. The ligands were used to generate the corresponding cobalt(II) chloride precatalysts **Co1**–**Co5**. The characterization procedure included single-crystal X-ray diffraction analysis for **Co3** and **Co4**. Activated with either MAO or MMAO, all the title complexes displayed high activity and thermal stability in ethylene polymerization up to 60 °C. The least sterically encumbered precatalyst **Co1** reached the peak activity of 1.00 × 10^7^ g PE mol^−1^(Co) h^−1^ with MAO at 60 °C and 7.89 × 10^6^ g PE mol^−1^(Co) h^−1^ with MMAO at 50 °C, which suggests high thermal stability of the active sites. Notably, the most sterically hindered precatalyst **Co3** has the propensity to generate polyethylene of the highest molecular weight. The catalytic system maintains good activity of 4.10 × 10^6^ g PE mol^−1^(Co) h^−1^ at the temperature as high as 80 °C. Therefore, we believe that it might be considered as a potential candidate for the industrial polymerization process, where the quest for novel precatalysts generating active and thermally stable active sites still encounters obstacles difficult to overcome.

## Figures and Tables

**Table 1:** Selected bond lengths (Å) and angles (°) for **Co3** and **Co4**.

	Co3	Co4
Bond Lengths (Å)	Molecule A	Molecule A
Co(1)–N(1)	2.043(5)	2.033(7)
Co(1)–N(2)	2.214(5)	2.188(6)
Co(1)–N(3)	2.228(5)	2.191(7)
Co(1)–Cl(1)	2.2481(18)	2.251(2)
Co(1)–Cl(2)	2.3047(18)	2.308(2)
N(2)–C(10)	1.437(7)	1.463(9)
N(3)–C(47)	1.453(8)	1.428(13)
N(1)–C(3)	1.345(7)	1.348(13)
N(1)–C(7)	1.338(7)	1.357(12)
N(2)–C(8)	1.286(7)	1.289(10)
N(3)–C(2)	1.274(7)	1.289(12)
**Bond Angles (°)**		
N(1)–Co(1)–N(2)	74.25(17)	73.90(3)
N(1)–Co(1)–N(3)	74.85(17)	74.10(3)
N(2)–Co(1)–N(3)	144.51(17)	142.10(3)
N(1)–Co(1)–Cl(1)	147.63(14)	150.90(2)
N(2)–Co(1)–Cl(1)	98.53(13)	99.28(18)
N(3)–Co(1)–Cl(1)	98.24(13)	98.80(2)
N(2)–Co(1)–Cl(2)	99.45(13)	99.87(18)
N(3)–Co(1)–Cl(2)	101.00(13)	102.76(18)
Cl(1)–Co(1)–Cl(2)	115.82(7)	113.91(9)
N(1)–Co(1)–Cl(2)	96.54(14)	95.10(2)
C(10)–N(2)–Co(1)	122.50(4)	124.4(5)
C(47)–N(3)–Co(1)	124.00(4)	124.80(7)

**Table 2:** Distance of the Co atom from the N1, N2, N3 plane, angles in the coordination sphere and the corresponding tau values (*τ_5_*) for **Co3** and **Co4.**

Complex	Molecule	Co distance, Å	*α,* °	*β,* °	*τ*_5_^a^
**Co3**	*A*	0.543	144.51	147.63	0.052
	*B*	0.513	141.15	154.04	0.215
**Co4**	*A*	0.532	142.10	150.90	0.147
	*B*	0.806	142.70	150.70	0.133

^a^ As defined by Equation (1).

**Table 3:** Optimization of the polymerization conditions for the **Co1**/MAO system *^a^*.

Entry	T, ^o^C	t, min	Al/Co	PE, g	Activity *^b^*	*M*_w_^c^	*M*_w_/*M*_n_^c^	*T*_m_, °C *^d^*
1	30	30	2000	3.51	4.68	4.50	2.80	135.6
2	40	30	2000	4.18	5.57	4.01	2.79	135.6
3	50	30	2000	4.82	6.43	3.05	3.61	135.5
4	60	30	2000	5.52	7.36	0.41	3.16	134.2
5	70	30	2000	5.04	6.72	0.30	2.58	131.8
6	80	30	2000	3.07	4.09	0.23	3.60	131.5
7	90	30	2000	1.04	1.39	0.20	5.18	130.7
8	60	30	1500	1.93	1.85	0.40	2.84	132.9
9	60	30	2500	5.83	7.77	0.43	3.22	132.7
10	60	30	2750	6.81	9.08	0.44	2.86	132.7
11	60	30	3000	7.51	10.0	0.52	2.45	132.6
12	60	30	3250	7.04	9.39	0.46	1.98	132.9
13	60	30	3500	5.76	7.68	0.38	2.50	132.6
14	60	5	3000	2.07	21.6	0.30	2.84	132.1
15	60	15	3000	4.25	11.3	0.50	3.45	133.1
16	60	45	3000	7.91	7.03	0.53	2.31	133.3
17	60	60	3000	8.72	5.81	0.60	2.88	132.8
18 *^e^*	60	30	3000	3.98	5.10	0.24	2.91	131.4
19 *^f^*	60	30	3000	0.64	0.85	0.02	1.82	123.1

*^a^* General conditions: 1.5 μmol of **Co1**, 100 mL toluene, 10 atm C_2_H_4_, unless indicated otherwise. *^b^*10^6^ g of PE mol^−1^(Co) h^−1^. ^c^ 10^5^ g mol^–1^, determined by GPC. *^d^* Determined by DSC. *^e^* 5 atm C_2_H_4_. *^f^* 1 atm C_2_H_4._

**Table 4:** Ethylene polymerization with (**Co1**–**Co5**)/MAO under the optimized conditions *^a^*.

Entry	Precatalyst	PE, g	Activity *^b^*	*M*_w_^c^	*M*_w_/*M*_n_^c^	*T_m_*, °C *^d^*
1	**Co1**	7.51	10.0	0.52	2.45	132.6
2	**Co2**	6.23	8.31	0.53	2.42	133.7
3	**Co3**	5.62	7.49	1.14	2.84	135.7
4	**Co4**	7.06	9.41	0.35	2.12	132.3
5	**Co5**	6.70	8.93	0.61	2.94	132.9

*^a^* General conditions: 1.5 μmol of precatalyst, 100 mL toluene, 10 atm C_2_H_4_, 60 °C, 30 min, Al/Co ratio of 3000. *^b^* 10^6^ g PE mol^−1^(Co) h^−1^. *^c^* 10^5^ g mol^–1^, determined by GPC. *^d^* Determined by DSC.

**Table 5:** Optimization of the polymerization conditions for the **Co1**/MMAO system *^a^*.

Entry	T, °C	T, min	Al/Co	PE, g	Activity *^b^*	*M*_w_ *^c^*	*M*_w_ */M_n_^c^*	*T_m_,* °C *^d^*
1	30	30	2000	3.91	5.21	3.80	3.70	135.8
2	40	30	2000	4.34	5.79	2.51	2.53	136.2
3	50	30	2000	4.71	6.28	1.98	2.65	135.8
4	60	30	2000	3.09	4.12	0.73	3.65	132.9
5	70	30	2000	2.80	3.73	0.36	3.02	134.6
6	80	30	2000	2.24	2.99	0.30	3.08	131.6
7	90	30	2000	1.42	1.89	0.25	2.91	131.3
8	50	30	1000	3.48	4.64	0.97	3.68	135.2
9	50	30	1250	5.24	6.99	2.02	3.43	135.4
10	50	30	1500	5.92	7.89	2.16	3.92	135.3
11	50	30	1750	5.03	6.71	2.38	3.64	135.7
12	50	30	2500	4.01	5.35	1.85	3.35	135.7
13	50	30	3000	3.76	5.01	1.61	2.12	134.9
14	50	5	1500	3.60	28.8	1.69	2.70	135.6
15	50	15	1500	4.65	12.4	1.75	2.83	135.5
16	50	45	1500	6.73	5.98	2.20	2.65	135.4
17	50	60	1500	8.64	5.76	2.25	3.89	135.3
18 *^e^*	50	30	1500	2.76	3.68	0.84	3.78	133.7
19 *^f^*	50	30	1500	0.35	0.47	0.08	2.36	128.7

*^a^* General conditions: 1.5 μmol of **Co1**, 100 mL toluene, 10 atm C_2_H_4_. *^b^* 10^6^ g of PE mol^−1^(Co) h^−1^. *^c^* 10^5^ g mol^–1^, determined by GPC. *^d^* Determined by DSC. *^e^* 5 atm C_2_H_4_. *^f^* 1 atm C_2_H_4._

**Table 6:** Ethylene polymerization with the (**Co1**–**Co5**)/MMAO at the optimized conditions *^a^*.

Entry	Precatalyst	PE, g	Activity *^b^*	*M*_w_^c^	*M*_w_/*M*_n_^c^	*T*_m_, °C ^d^
1	**Co1**	5.92	7.89	2.38	3.92	135.3
2	**Co2**	5.53	7.37	5.78	6.62	135.7
3	**Co3**	5.04	6.72	6.46	3.17	136.8
4	**Co4**	5.74	7.65	3.70	5.79	136.8
5	**Co5**	5.42	7.23	3.92	3.19	136.7

*^a^* General conditions: 1.5 μmol of precatalyst, 100 mL toluene, 10 atm C_2_H_4_, 50 °C, 30 min, Al/Co ratio of 1500. *^b^* 10^6^ g PE mol^−1^(Co) h^−1^_._ *^c^* 10^5^ g mol^–1^, determined by GPC. *^d^* Determined by DSC.

**Table 7:** Crystal data and structure refinement for the **Co3** and **Co4** complexes.

Identification Code	Co3	Co4
CCDC number	1905876	1905877
Empirical formula	2(C_58_H_56_Cl_2_CoN_3_)	2(C_55_H_50_Cl_2_CoN_3_)
Formula weight	924.88	883.31
Temperature (K)	173.1500	173.1500
Wavelength (Å)	0.71073	0.71073
Crystal system	monoclinic	orthorhombic
Space group	*P21/c*	*Pna21*
a (Å)	26.6132(7)	16.5906(3)
b (Å)	24.0122(6)	12.1091(3)
c (Å)	17.1072(4)	49.9111(9)
α (°)	90	90
β (°)	105.153(3)	90
γ (°)	90	90
Volume (Å^3^)	10552.1(5)	10027.0(4)
Z	8	4
D calcd (g/cm^3^)	1.164	1.170
*μ* (mm^-1^)	0.464	0.486
*F* (000)	3888.0	3700.0
Crystal size (mm^3^)	0.376 × 0.105 × 0.082	0.476 × 0.118 × 0.056
*θ* range (°)	2.994 to 50	3.264 to 55
Limiting indexes	−31 ≤ h ≤ 31, −28 ≤ k ≤ 28, −20 ≤ l ≤ 20	−21 ≤ h ≤ 21, −15 ≤ k ≤ 15, −64 ≤ l ≤ 64
No. of rflns collected	121754	135076
No. of unique rflns [R(int)]	18602 (0.1031)	23020 (0.0956)
Completeness to *θ* (%)	1.00	1.98
Data/restraints/parameters	18602/445/1167	23020/177/1149
Goodness of fit on *F*^2^	1.041	0.988
Final R indexes [*I* > = 2σ (*I*)]	R_1_ = 0.0965 wR_2_ = 0.2394	R_1_ = 0.0691, wR_2_ = 0.1588
R indexes (all data)	R_1_ = 0.1397 wR_2_ = 0.2729	R_1_ = 0.1134, wR_2_ = 0.1841
Largest diff. peak and hole (e Å^–3^)	1.36/–0.82	0.60/–0.28

## Supplementary Materials

The following are available online at https://www.mdpi.com/1420-3049/24/10/2007/s1, Figure S1: ^1^H NMR spectrum of **Co1** in CD_2_Cl_2_ at room temperature. Figure S2: ^1^H NMR spectrum of **Co2** in CD_2_Cl_2_ at room temperature. Figure S3: ^1^H NMR spectrum of **Co3** in CD_2_Cl_2_ at room temperature. Figure S4: ^1^H NMR spectrum of **Co4** in CD_2_Cl_2_ at room temperature. Figure S5: ^1^H NMR spectrum of **Co5** in CD_2_Cl_2_ at room temperature. Figure S6: GPC curves of the obtained polyethylene (**a**); activity and *M*_w_ as a function of reaction temperature (**b**) for the **Co1**/MMAO system (Table 5, entries 1 – 7). Figure S7: GPC curves of the obtained polyethylene (**a**); activity and *M*_w_ as a function of Al/Co ratio (**b**) for the **Co1**/MMAO system (Table 5, entries 3 and 8 – 13). Figure S8: GPC curves of the obtained polyethylene (**a**); activity and *M*_w_ as a function of run time (**b**) for the **Co1**/MMAO system (Table 5, entries 10 and 14 – 17). Figure S9: GPC curves of the obtained polyethylene (**a**); activity and *M*_w_ for different precatalysts (**b**) at the optimized reaction conditions with MMAO as cocatalyst (Table 6, entries 1 – 5). Figure S10: GPC curves of the obtained polyethylene (**a**); activity and *M*_w_ as a function of ethylene pressure (**b**) at the optimized reaction conditions for the **Co1**/MMAO system (Table 5, entries 10, 18 and 19). Figure S11: The ^1^H NMR spectrum of the polyethylene obtained with **Co1**/MMAO (Table 5, entry 10). Figure S12: The ^13^C NMR spectrum of the polyethylene obtained with **Co1**/MMAO (Table 5, entry 10). Table S1: The selected bond lengths (Å) and angles (^o^) for the *B* molecules of **Co3** and **Co4**.
