# Supplementary material for: Activity and Thermal Stability of Cobalt(II)-Based Olefin Polymerization Catalysts Adorned with Sterically Hindered Dibenzocycloheptyl Groups
Source: Molecules. 2019 May 25;24(10):2007. doi: 10.3390/molecules24102007 (PMC6572594; doi:10.3390/molecules24102007)
Supplement: Supplementary file 1 [file molecules-24-02007-s001.pdf]

Supporting information

for

# Activity and Thermal Stability of Cobalt(II)-Based Olefin Polymerization Catalysts Adorned with Sterically Hindered Dibenzocycloheptyl Groups

**Muhammad Zada,[1,2] Liwei Guo,[1,3] Yanping Ma,[1] Wenjuan Zhang,[3,*] Zygmunt Flisak,[1,4,*] Yang Sun,[1] Wen-Hua Sun [1,2,*]**

[1] Key Laboratory of Engineering Plastics and Beijing National Laboratory for Molecular Science, Institute of Chemistry, Chinese Academy of Sciences, Beijing 100190, China. mzada17@iccas.ac.cn (M.Z.); mrliwei_guo@163.com (L.G.); myanping@ iccas.ac.cn (Y.M.); sy0471103 iccas.ac.cn (Y.S.)

[2] CAS Research/Education Center for Excellence in Molecular Sciences, University of Chinese Academy of Sciences, Beijing 100049, China.

[3] Beijing Key Laboratory of Clothing Materials R&D and Assessment, Beijing Engineering Research Center of Textile Nanofiber, School of Materials Science and Engineering, Beijing institute of Fashion Technology, Beijing 100029, China. zhangwj@bift.edu.cn (W.Z.)

[4] Faculty of Chemistry, University of Opole, Oleska 48, 45-052 Opole, Poland. zgf@uni.opole.pl (Z.F.)

* Correspondence: zhangwj@bift.edu.cn (W.Z.); zgf@uni.opole.pl (Z.F.); whsun@iccas.ac.cn (W.-H.S.); Tel.: +86-10-6255-7955 (W.-H.S.)

| **Table of Contents** | Page |
|---|---|
| 1. The $^{1}$H NMR spectra of precatalysts (**Co1** – **Co5)** | S2 |
| 2. GPC curves of the obtained polyethylene (**a**); activity and $M_w$ as a function of reaction temperature, Al/Co ratio and run time (**b**) for the **Co1**/MMAO system | S4 |
| 3. GPC curves of the obtained polyethylene (**a**); activity and $M_w$ for different precatalysts (**b**) at the optimized reaction conditions with MMAO as cocatalyst | S6 |
| 4. GPC curves of the obtained polyethylene (**a**); activity and $M_w$ as a function of ethylene pressure (**b**) at the optimized reaction conditions for the **Co1**/MMAO system | S6 |
| 5. The $^{1}$H and $^{13}$C NMR spectra of the polyethylene obtained with **Co1**/MMAO | S7 |
| 6. The selected bond lengths (Å) and angles (°) for the *B* molecules of **Co3** and **Co4** | S8 |

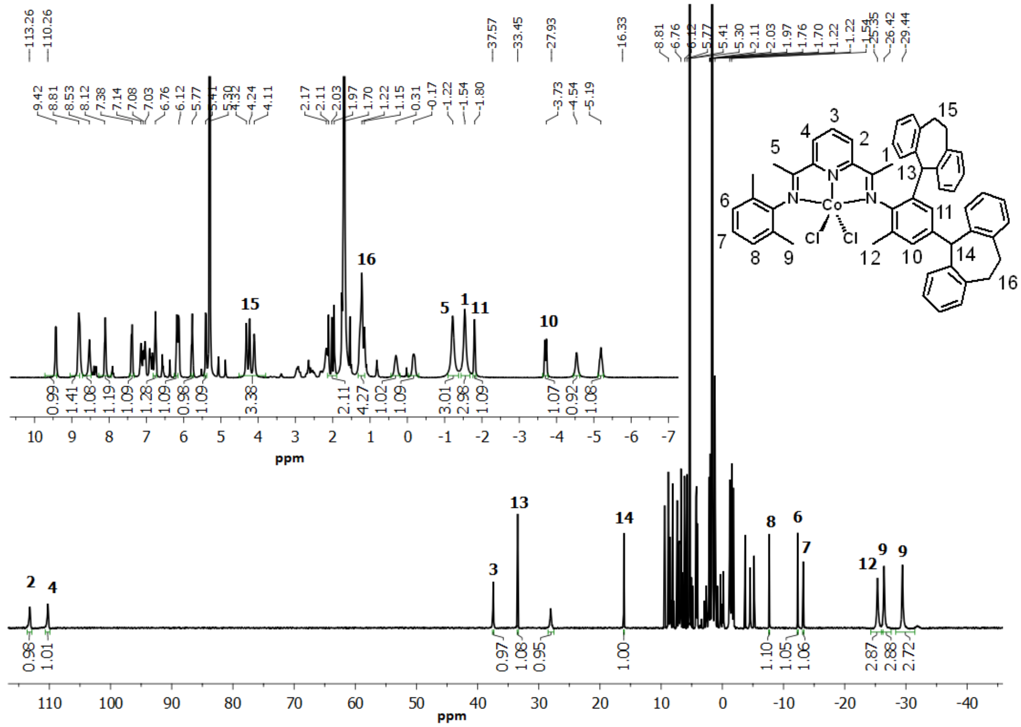

**Figure S1** $^{1}$H NMR spectrum of **Co1** in $CD_2Cl_2$ at room temperature.

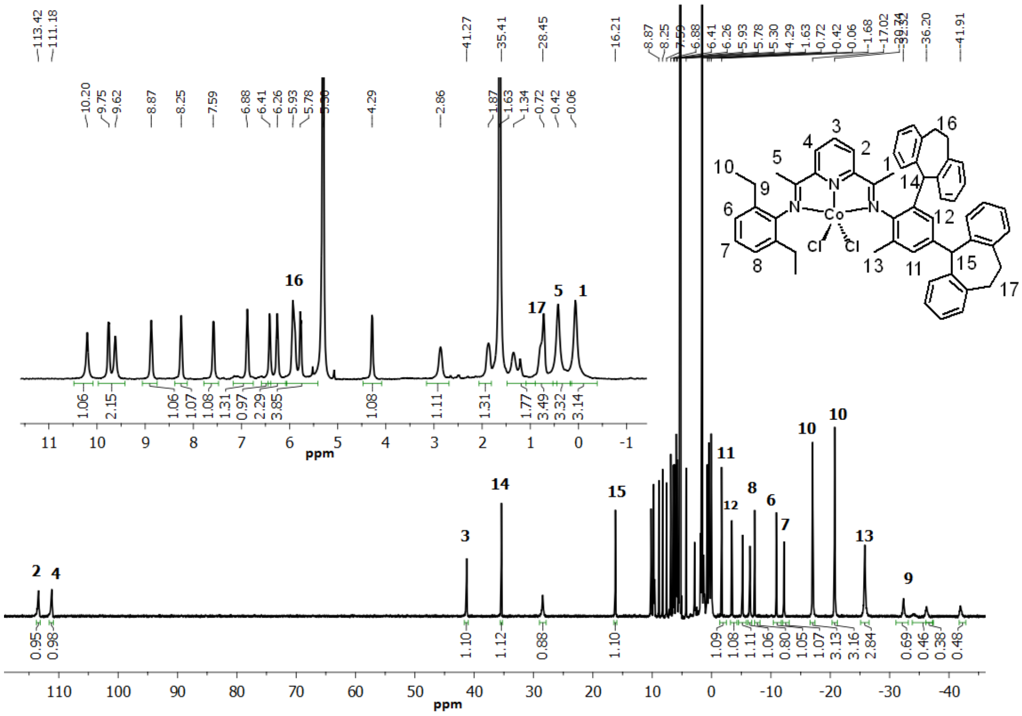

**Figure S2** $^{1}$H NMR spectrum of **Co2** in $CD_2Cl_2$ at room temperature.

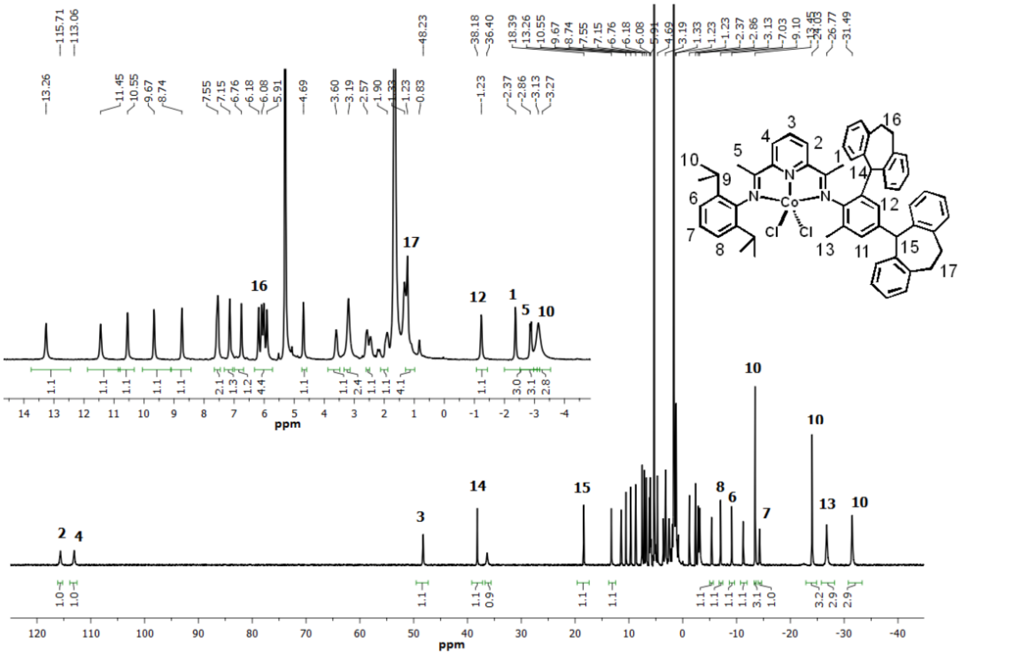

**Figure S3** $^{1}$H NMR spectrum of **Co3** in $CD_2Cl_2$ at room temperature.

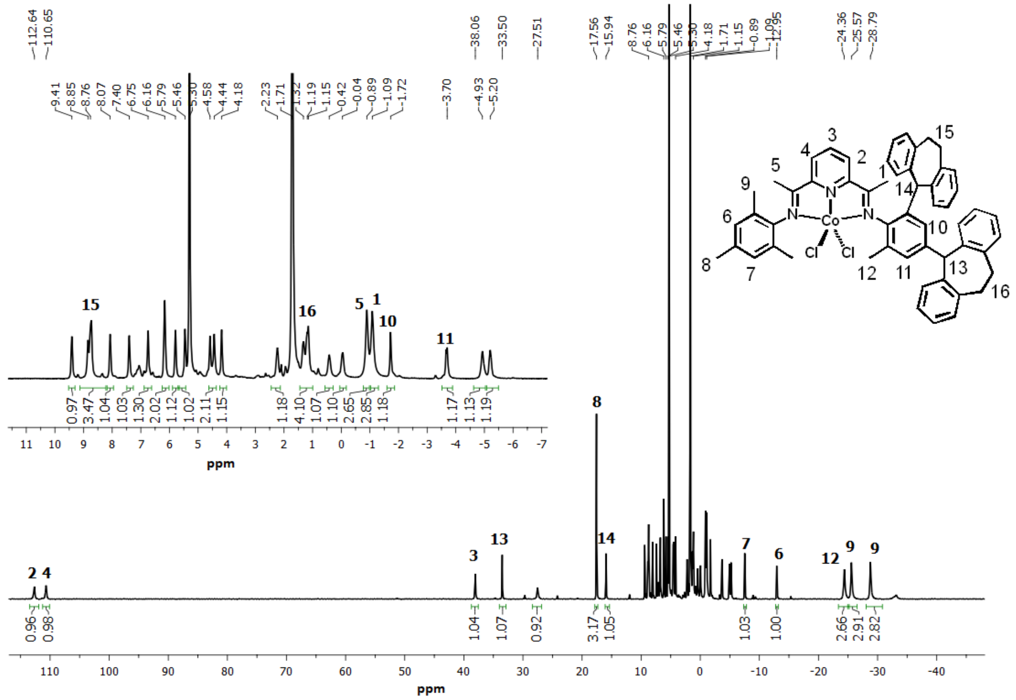

**Figure S4** $^{1}$H NMR spectrum of **Co4** in $CD_2Cl_2$ at room temperature.

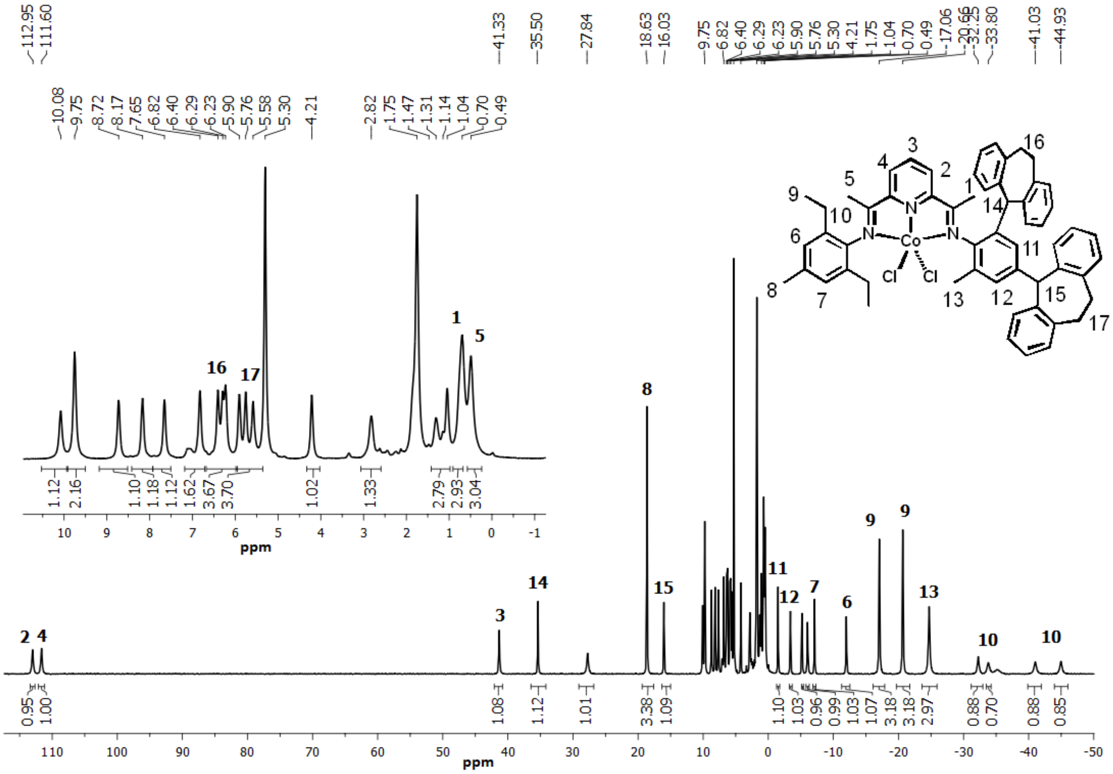


**Figure S5** $^{1}H$ NMR spectrum of **Co5** in $CD_2Cl_2$ at room temperature.

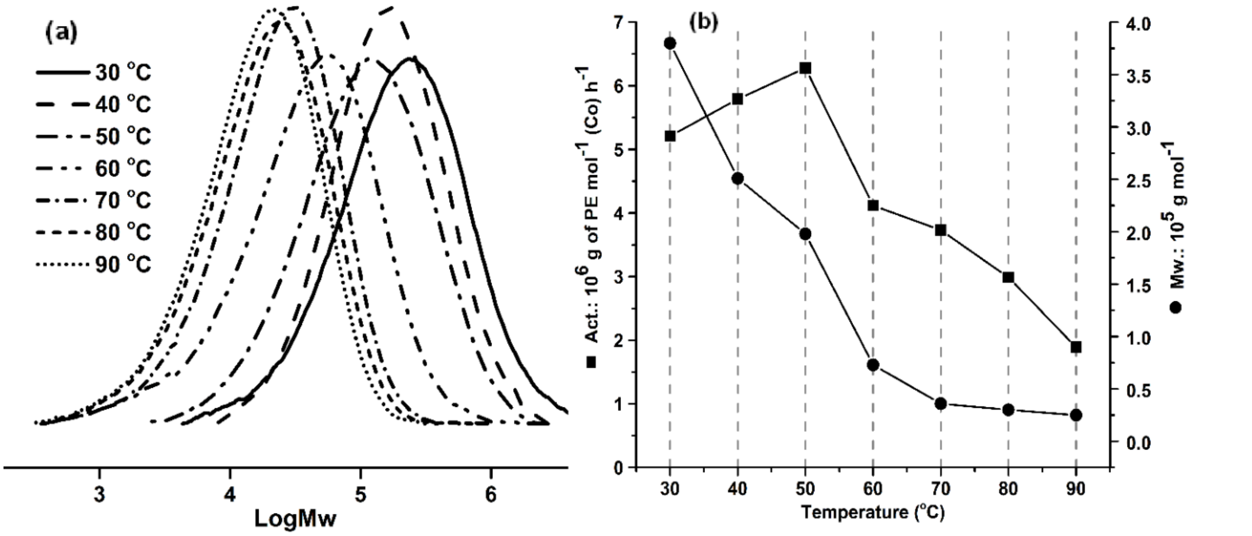


**Figure S6** GPC curves of the obtained polyethylene (**a**); activity and $M_w$ as a function of reaction temperature (**b**) for the **Co1**/MMAO system (Table 5, entries 1 – 7).

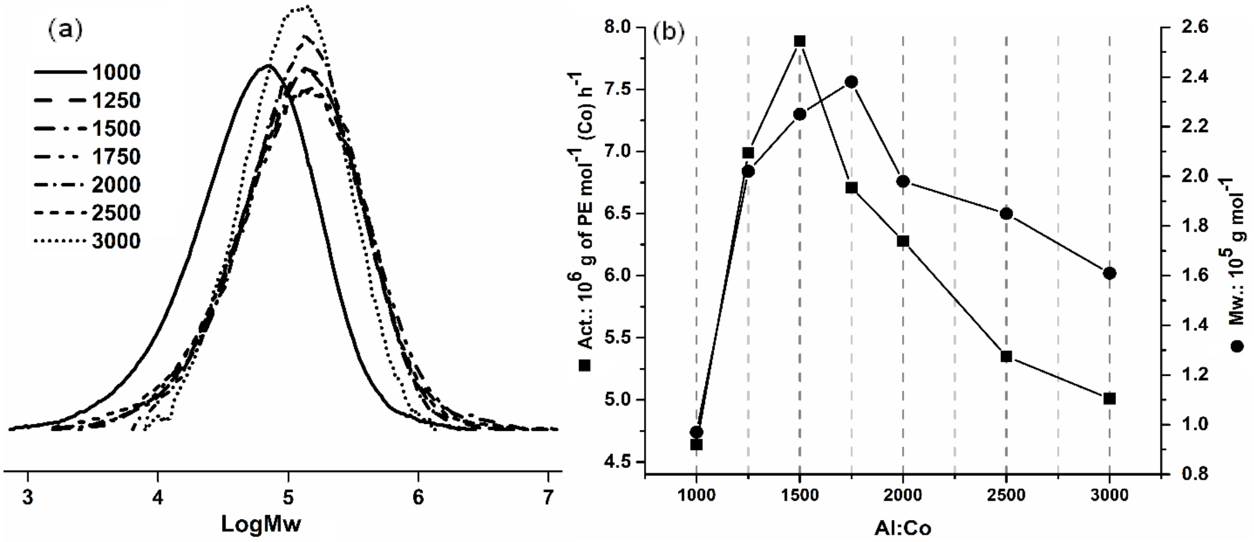


**Figure S7** GPC curves of the obtained polyethylene (**a**); activity and $M_w$ as a function of Al/Co ratio (**b**) for the **Co1**/MMAO system (Table 5, entries 3 and 8 – 13).

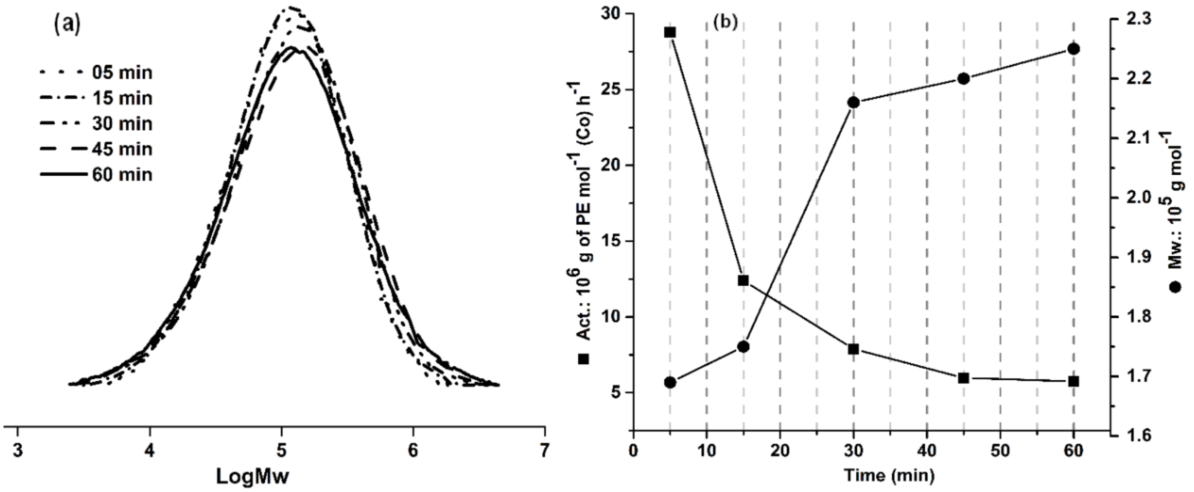


**Figure S8** GPC curves of the obtained polyethylene (**a**); activity and $M_w$ as a function of run time (**b**) for the **Co1**/MMAO system (Table 5, entries 10 and 14 – 17).

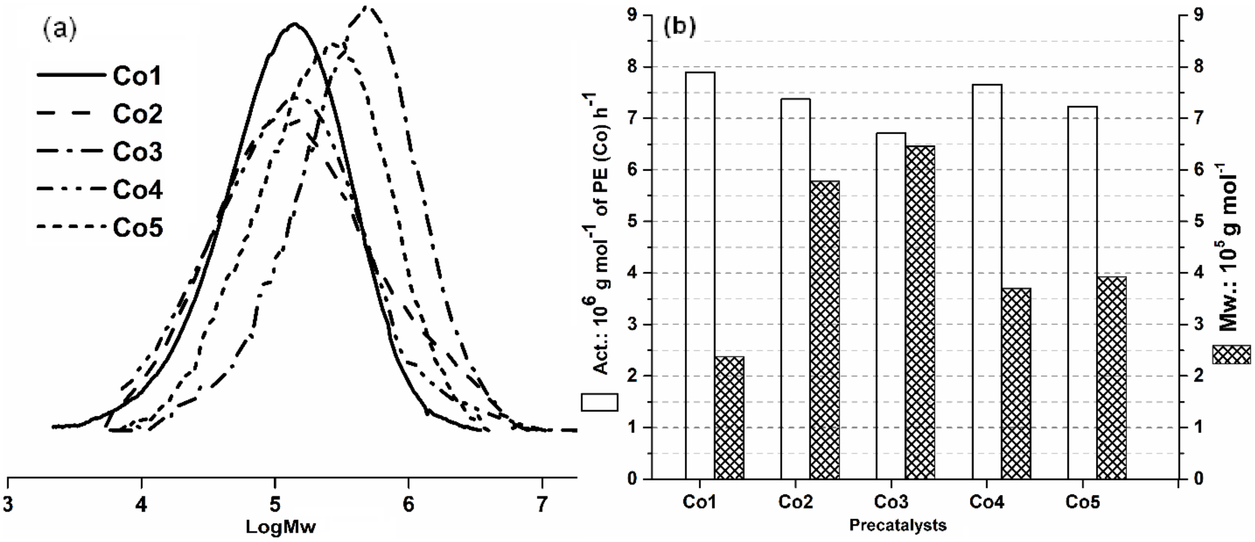


**Figure S9** GPC curves of the obtained polyethylene (**a**); activity and $M_w$ for different precatalysts (**b**) at the optimized reaction conditions with MMAO as cocatalyst (Table 6, entries 1 – 5).

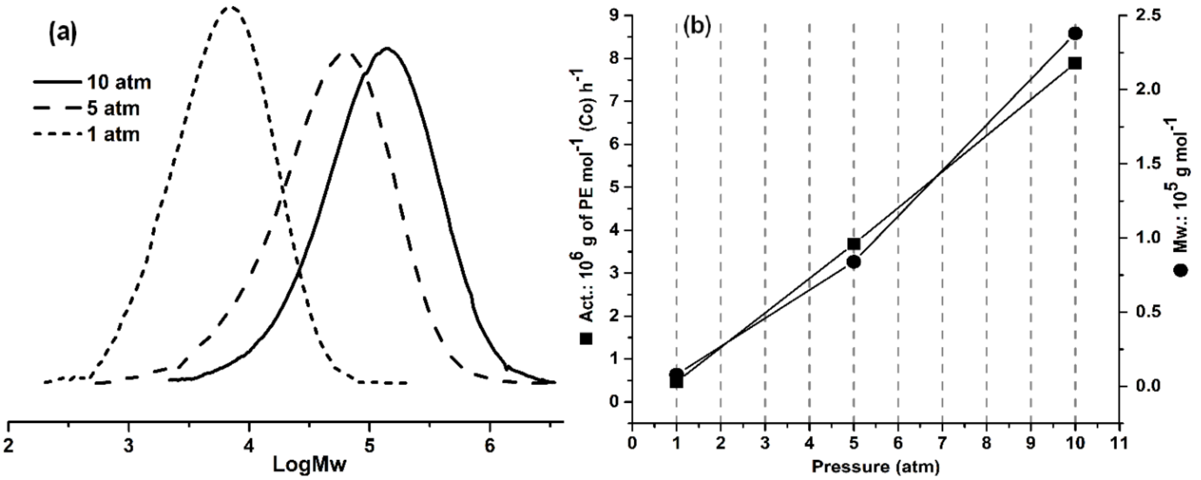


**Figure S10** GPC curves of the obtained polyethylene (**a**); activity and $M_w$ as a function of ethylene pressure (**b**) at the optimized reaction conditions for the **Co1**/MMAO system (Table 5, entries 10, 18 and 19).

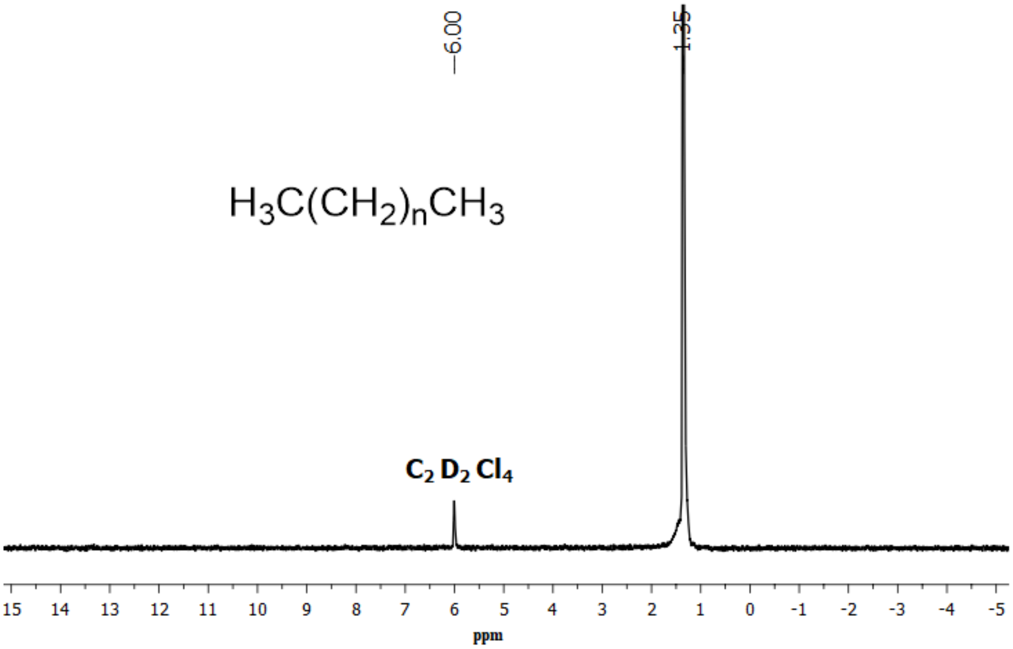


**Figure S11** The $^{1}$H NMR spectrum of the polyethylene obtained with **Co1**/MMAO (Table 5, entry 10).

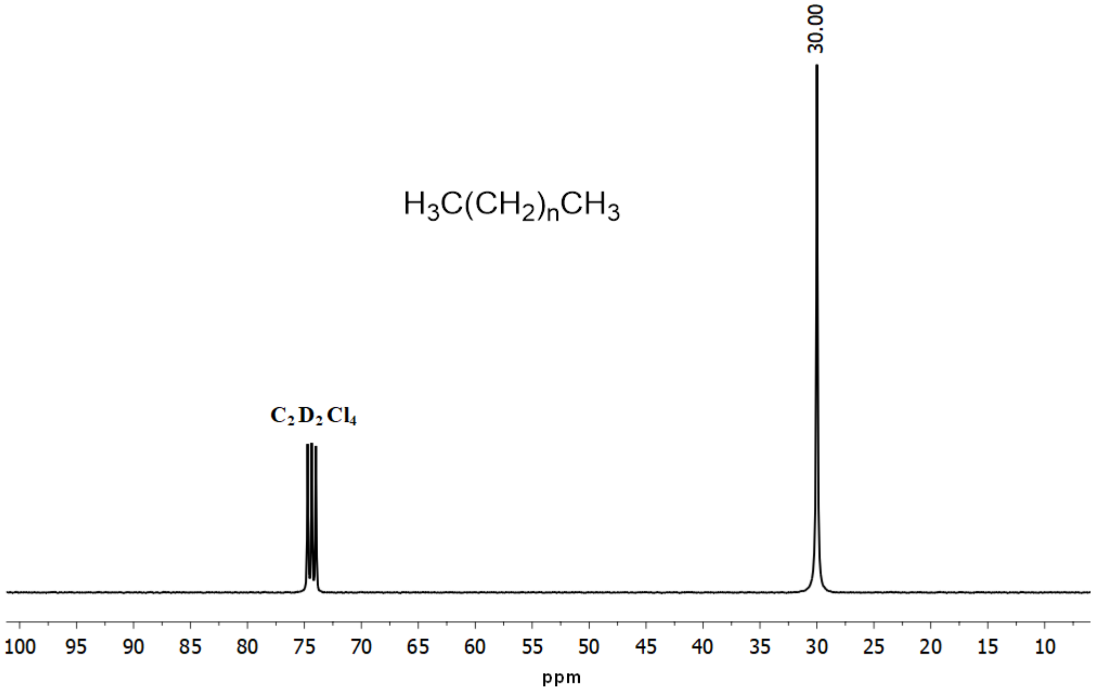


**Figure S12** The $^{13}$C NMR spectrum of the polyethylene obtained with **Co1**/MMAO (Table 5, entry 10).

**Table S1** The selected bond lengths (Å) and angles (º) for the *B* molecules of **Co3** and **Co4**

| | **Co3** | **Co4** |
|---|---|---|
| Bond Lengths (Å) | *Molecule B* | *Molecule B* |
| Co(1)–N(1) | 2.062(4) | 2.059(7) |
| Co(1)–N(2) | 2.216(4) | 2.177(7) |
| Co(1)–N(3) | 2.230(5) | 2. 212(7) |
| Co(1)–Cl(1) | 2.2572(16) | 2.265(2) |
| Co(1)–Cl(2) | 2.2957(16) | 2.298(2) |
| N(2)–C(10) | 1.441(7) | 1.437(10) |
| N(3)–C(47) | 1.448(8) | 1.435(12) |
| N(1)–C(3) | 1.349(7) | 1.335(13) |
| N(1)–C(7) | 1.320(7) | 1.317(12) |
| N(2)–C(8) | 1.288(7) | 1.272(10) |
| N(3)–C(2) | 1.295(8) | 1.289(12) |
| Bond Angles (º) | | |
| N(1)–Co(1)–N(2) | 73.47(17) | 74.40(3) |
| N(1)–Co(1)–N(3) | 74.46(18) | 74.40(3) |
| N(2)–Co(1)–N(3) | 141.15(17) | 142.70(3) |
| N(1)–Co(1)–Cl(1) | 154.04(13) | 150.70(2) |
| N(2)–Co(1)–Cl(1) | 99.00(12) | 98.34(17) |
| N(3)–Co(1)–Cl(1) | 100.26(15) | 98.90(2) |
| N(2)–Co(1)–Cl(2) | 102.35(12) | 100.44(18) |
| N(3)–Co(1)–Cl(2) | 101.25(15) | 102.23(19) |
| Cl(1)–Co(1)–Cl(2) | 111.86(6) | 114.16(9) |
| N(1)–Co(1)–Cl(2) | 94.08(13) | 95.10(2) |
| C(10)–N(2)–Co(1) | 124.20(3) | 125.40(5) |
| C(47)–N(3)–Co(1) | 125.20(4) | 123.70(6) |
